# Determinants of C_16_-Ceramide Binding to p53

**DOI:** 10.3390/biom16091343

**Published:** 2026-09-15

**Authors:** Madeline S. Childress, Kristen A. Jeffries, Sergey A. Krupenko, Natalia I. Krupenko

**Affiliations:** 1 Nutrition Research Institute, UNC-Chapel Hill, Kannapolis, NC 28081, USA; madeline_childress@unc.edu (M.S.C.); jeffries.kaj@gmail.com (K.A.J.); 2 Department of Nutrition, UNC-Chapel Hill, Chapel Hill, NC 27599, USA; sergey_krupenko@unc.edu

**Keywords:** sphingolipid signaling, C_16_-ceramide, p53 activation, mutant p53, ER stress, CerS6

## Abstract

The tumor suppressor p53 coordinates cellular stress responses, but underlying mechanisms remain incompletely understood. We previously demonstrated that, in response to metabolic stress, C_16_-ceramide produced by ceramide synthase 6 (CerS6) directly binds to p53, thus preventing its MDM2-mediated degradation and promoting p53 activation. Here, we investigated the structural requirements and functional consequences of ceramide binding to p53. Using a panel of p53 mutants, including naturally occurring oncogenic variants, we characterized the ceramide-binding interface of p53 and the role of amino acid substitutions within this region in metabolic stress signaling. We found that disruption of ceramide binding impaired stress-induced p53–CerS6 interaction at the endoplasmic reticulum (ER), attenuated induction of p53 target genes, and reduced cellular sensitivity to stress. Of note, certain cancer-associated p53 mutants retained ceramide binding and the ability to activate stress responses. These studies were further extended to monitoring the p53–CerS6 interaction on the ER and associated membrane aggregation using fluorescence techniques. We showed that metabolic stress-induced ER remodeling was distinct from the canonical UPR. Overall, our study defines the ceramide-binding surface within the p53 DNA-binding domain and provides novel insight into the functional role of the ceramide–p53 interaction.

## 1. Introduction

The transcription factor and major tumor suppressor p53 is a central regulator of cellular stress responses and a primary barrier to malignant transformation [1,2]. In unstressed cells, p53 protein is maintained at low levels primarily through MDM2-mediated ubiquitination and proteasomal degradation [3,4]. In response to diverse cellular insults, including DNA damage, oncogenic signaling, and metabolic perturbation, p53 coordinates transcriptional programs that determine whether cells undergo cell-cycle arrest, senescence, repair, or apoptosis [5]. Through these activities, p53 preserves genomic integrity and suppresses tumor development [6]. Consistent with this essential role, *TP53,* which encodes the p53 protein, is the most frequently mutated gene in human cancer, and disruption of p53 signaling represents one of the most common events in tumor progression [7].

In parallel, bioactive sphingolipids have emerged as critical mediators of cellular stress signaling [8,9]. Ceramides, central intermediates of sphingolipid metabolism, accumulate in response to chemotherapeutic agents, heat stress, and nutrient deprivation and are widely associated with growth arrest and apoptosis [10,11]. Ceramide synthesis is catalyzed by six ER-resident ceramide synthases (CerS1–6), each generating ceramides with defined acyl-chain lengths that exhibit nonredundant biological functions [12]. Among these species, CerS6-derived C_16_-ceramide has been repeatedly linked to proapoptotic and tumor-suppressive responses across multiple cell types [13]. Although both CerS5 and CerS6 can generate C_16_-ceramide, CerS6 was identified as the primary ceramide synthase mediating stress-induced p53 interaction and activation [14]. We previously demonstrated that C_16_-ceramide directly binds to p53, protects it from MDM2-mediated degradation, and promotes p53-dependent cell death [15]. Interestingly, p53 is a transcriptional activator of CerS6 expression, thereby increasing the generation of C_16_-ceramide [16]. This bilateral relationship between CerS6-derived C_16_-ceramide and p53 creates a feed-forward signaling loop that is activated in response to metabolic stress.

Within this signaling mechanism, transactivation of CerS6 by p53 is a canonical regulatory element, since p53 serves as a transcriptional regulator for hundreds of genes [17]. The mechanisms underlying p53 activation by C_16_-ceramide, however, remain incompletely understood. We have recently reported that direct interaction between p53 and CerS6 at the ER membrane is a key event that allows the direct channeling of CerS6-generated C_16_-ceramide to the DNA-binding domain (DBD) of p53 [18].

Importantly, the DBD not only provides the binding site for ceramide but also dictates recognition of specific ceramide acyl-chain length. Furthermore, it is solely responsible for mediating the interaction with CerS6. Unlike many tumor suppressors that are inactivated through deletion or truncation, *TP53* most commonly acquires missense mutations within the DBD [19]. These mutations cluster at recurrent hotspot residues and generally disrupt either structural stability or DNA contacts [20,21]. Although mutant p53 proteins are typically characterized by the loss of transcriptional activity, increasing evidence suggests that at least some mutant forms retain partial biochemical function and can be structurally or functionally rescued under specific conditions [22,23]. These observations raise the possibility that regulation of p53 may involve additional mutation-sensitive mechanisms operating within the DBD.

Our previous study identified Ser240 and Ser241, which reside within the DBD cancer mutations cluster, as direct C_16_-ceramide contact residues [15]. Notably, Ser240 lies within the p53 global suppressor motif, a region in which second-site mutations may restore stability and function to a broad range of destabilized p53 mutants [20,21]. This arrangement suggests that ceramide binding may not only influence p53 degradation but also modulate protein activity through a ceramide-responsive regulatory surface within the DBD. The integrity of this surface could determine the ability of p53 to recognize C_16_-ceramide and respond to metabolic stress. Many tumor-acquired mutations occur within the same DBD surface that mediates ceramide interaction and therefore could disrupt ceramide binding independently of canonical DNA-binding defects. Loss of ceramide recognition is predicted to uncouple metabolic stress signaling from p53 stabilization and apoptotic activation and may provide tumor cells with a selective survival advantage. Indeed, the germline S241F mutation, a pathogenic event leading to loss of tumor-suppression function, is linked to increased cancer susceptibility (Li–Fraumeni syndrome) [24], while the S240R substitution is oncogenic and affects p21-linked cell-cycle regulation [25]. At present, it is not clear which clinically relevant p53 mutants retain C_16_-ceramide binding and whether ceramide-binding status correlates with p53 pathway activation, interaction with CerS6 at the ER membrane, and cellular sensitivity to metabolic stress. Recent molecular dynamics simulations have begun to examine the structural basis of this interaction [26], but experimental validation remains limited.

In the present study, we investigated how mutations within the hotspot cluster of the p53 DBD, located in the vicinity of the ceramide-interacting interface, affect p53–CerS6 interaction at the ER, C_16_-ceramide binding, and cellular responses to metabolic stress. We have used targeted amino acid substitutions, naturally occurring p53 mutant cell lines, transient expression of hotspot mutants, and bimolecular fluorescence complementation (BiFC) assays to assess the relationship between p53 mutants and ceramide signaling. To this end, we also determined whether ceramide-binding status influences the induction of specific p53 targets (p21, PUMA, and CerS6) as well as cellular sensitivity to stress. Our study further distinguished p53–CerS6-dependent ER remodeling during metabolic stress from canonical unfolded protein response (UPR) signaling.

## 2. Materials and Methods

### 2.1. Cell Culture

A549 human lung carcinoma cells (American Type Culture Collection Manassas, VA, USA; ATCC CCL-185), PC-3 human prostate carcinoma cells (ATCC CRL-1435), NCI-H1770 (ATCC CRL-5893), NCI-H596 (ATCC HTB-178), BT-549 (ATCC HTB-122), NCI-H1975 (ATCC CRL-5908), NCI-H510A (ATCC HTB-184), and DLD-1 (ATCC CCL-221) were obtained from the American Type Culture Collection (ATCC). A549 cells with stable RNAi-mediated knockdown of p53 (A549/p53-shRNA) were generated previously in our laboratory [27]. A549, A549/p53-shRNA, PC-3, NCI-H1770, NCI-H596, NCI-H1975, and DLD-1 cells were maintained in RPMI-1640 medium (Gibco, Thermo Fisher Scientific, Waltham, MA, USA; Cat. #11875-093) supplemented with 10% (*v*/*v*) fetal bovine serum (FBS; Gibco, Cat. #26140079), 2 mM L-glutamine (Gibco, Cat. #25030081), 1 mM sodium pyruvate (Gibco, Cat. #11360070), and 1× antibiotic–antimycotic solution (PSN; Gibco, Cat. #15640055). BT-549 cells were cultured in the same medium supplemented with 0.023 U/mL insulin. A549/p53-shRNA cells were cultured under identical conditions with the addition of 0.5 μg/mL puromycin (Gibco, Cat. #A1113803). NCI-H510A cells were maintained in Ham’s F-12 medium supplemented with 10% (*v*/*v*) FBS and 1× PSN. Cells were maintained at 37 °C in a humidified atmosphere containing 5% CO_2_ and were routinely used between passages 5 and 20.

### 2.2. Reagents

Methotrexate (MTX; Sigma-Aldrich, St. Louis, MO, USA; Cat. #454126) was dissolved in sterile water as a 10 mM stock solution. Pyridinium C_16_-ceramide (PyrC_16_; *D*-erythro-2-*N*-[16′-(1″-pyridinium)-hexadecanoyl]-sphingosine bromide) and PyrC6 (*D*-erythro-2-*N*-[6′-(1″-pyridinium)-hexanoyl]-sphingosine bromide) were synthesized by the MUSC Lipidomics Shared Resource/Synthetic Core (Charleston, SC, USA). Gemcitabine (Millipore Sigma, Burlington, MA, USA; Cat. #50-459-40001), doxorubicin (Fisher Bioreagents, Thermo Fisher Scientific, Waltham, MA, USA; Cat. #BP2516-10), daunorubicin (Tocris Bioscience, Bristol, UK; Cat. #1467), etoposide (Cayman Chemical, Ann Arbor, MI, USA; Cat. #12092), actinomycin D (Alfa Aesar, Ward Hill, MA, USA; Cat. #J67160), Nutlin-3 (Tocris, Cat. #3984), MG-132 (Tocris, Cat. #1748), tunicamycin (Millipore Sigma, Cat. #T7765-1MG), thapsigargin (Invitrogen, Thermo Fisher Scientific, Waltham, MA, USA; Cat. #T7458), and dithiothreitol (DTT; Thermofisher, Thermo Fisher Scientific, Waltham, MA, USA; Cat. #R0861) were obtained from commercial sources.

Unless otherwise indicated, cells were treated in complete growth medium. Treatments were performed at the concentrations and durations indicated in the corresponding figure legends and included MTX (100 nM for 4 h or 10 nM for 24 h), PyrC_16_ (5 μM for 18 or 24 h), tunicamycin (10 μg/mL for 4 h or 4 μg/mL for 24 h), thapsigargin (1 μM for 4 h or 0.2 μM for 24 h), DTT (4 mM for 4 h), gemcitabine (200 nM for 24 h), doxorubicin (700 nM for 24 h), daunorubicin (2 μM for 24 h), etoposide (100 μM for 24 h), actinomycin D (50 nM for 24 h), Nutlin-3 (30 μM for 24 h), or MG-132 (10 μM for 24 h).

### 2.3. Plasmid Construction and Site-Directed Mutagenesis

A human p53 expression plasmid was used to generate all p53 variants examined in this study. Point mutations were introduced into full-length p53 by the QuikChange Lightning Site-Directed Mutagenesis Kit (Agilent Technologies, Santa Clara, CA, USA; Cat. #210518) according to the manufacturer’s instructions. Mutagenic primers were synthesized by Eurofins Genomics, and all constructs were verified by Sanger sequencing (Eurofins Genomics, Louisville, KY, USA).

For bimolecular fluorescence complementation (BiFC) experiments, the Venus-based pCS2/V1-p53 and pCS2/MDM2-V2 constructs were obtained from Prof. Cecilia Rodrigues [28]. The CerS6-V2 construct was generated by cloning the human CerS6 coding sequence, previously described by Senkal et al. [29], into the corresponding pCS2/MDM2-V2 recipient vector after removal of the MDM2 coding sequence to generate an in-frame C-terminal Venus V2 fusion protein. Mutant V1-p53 constructs were generated by site-directed mutagenesis as described above and verified by Sanger sequencing.

### 2.4. Transient Transfection

Cells were seeded the day before transfection to achieve approximately 50–80% confluence at the time of transfection. Transient transfections were performed in antibiotic-free medium using Lipofectamine 3000 (Invitrogen, Thermo Fisher Scientific, Cat. #L3000008) according to the manufacturer’s instructions. Equal amounts of plasmid DNA were used for all single-plasmid transfections. For BiFC experiments, equal amounts of V1- and V2-fusion plasmids were co-transfected. Culture medium was replaced 6 h after transfection, and cells were allowed to recover overnight. Cells were harvested directly or subjected to the indicated treatments 24 h after transfection.

### 2.5. Protein Extraction for Ceramide Membrane-Binding Assays

Whole-cell lysates used for ceramide membrane-binding assays were prepared using immunoprecipitation (IP) buffer containing 20 mM Tris-HCl (pH 7.4), 150 mM NaCl, and 1 mM DTT supplemented immediately before use with Halt™ Protease and Phosphatase Inhibitor Cocktail (Thermo Fisher Scientific, Cat. #78442). Lysates were clarified by centrifugation at 16,000× *g* for 15 min at 4 °C, and protein concentrations were determined using the Pierce BCA Protein Assay Kit (Thermo Fisher Scientific, Cat. #23225).

### 2.6. Ceramide Membrane-Binding Assay

PyrC_16_ and PyrC_6_ were dissolved in DMSO as 10 mM stock solutions. Following methanol activation, equilibration in TBS, and removal of excess liquid using filter paper, equal volumes (0.5 μL) containing the indicated amounts of lipid were spotted onto 0.45 μm PVDF membranes (Thermo Fisher Scientific, Cat. #88518). DMSO alone served as the 0 μg control. Membranes were air-dried for 2–3 h, blocked for 1 h at room temperature with 3% BSA in TBST, and incubated overnight at 4 °C with clarified whole-cell lysates (1.5 mL, 1 mg/mL total protein) on a rocking platform. Membranes were washed three times for 10 min with 0.1% TBST and incubated with an in-house rabbit anti-p53 antibody (1:2000) for 1.5 h at room temperature. Following three additional washes, membranes were incubated for 1 h at room temperature with HRP-conjugated donkey anti-rabbit IgG (GE Healthcare Life Sciences, Chicago, IL, USA; Cat. #NA934; 1:5000). Membranes were washed again and developed using Immobilon Forte Western HRP Substrate (Millipore, Cat. #WBLUF0500). Chemiluminescent signals were acquired using an Odyssey Fc Imaging System (LI-COR Biosciences, Lincoln, NE, USA). PyrC_6_, which does not bind p53, served as a negative lipid control.

### 2.7. Western Blot Analysis

For immunoblotting, cells were lysed in RIPA buffer (Thermo Fisher Scientific, Cat. #89901) supplemented immediately before use with Halt™ Protease and Phosphatase Inhibitor Cocktail (Thermo Fisher Scientific, Cat. #78442). Lysates were clarified by centrifugation at 16,000× *g* for 15 min at 4 °C, and protein concentrations were determined using the Pierce BCA Protein Assay Kit (Thermo Fisher Scientific, Cat. #23225).

Whole-cell lysates were mixed with Laemmli sample buffer containing β-mercaptoethanol and heated at 100 °C for 5 min before electrophoresis. Samples prepared for CerS6 detection were incubated at room temperature for 15 min instead of boiling. Proteins were separated on homemade SDS-polyacrylamide gels using Tris-Glycine-SDS Running Buffer (Bio-Rad Laboratories, Hercules, CA, USA; Cat. #1610772) and transferred to methanol-activated 0.45 μm PVDF membranes (Thermo Fisher Scientific, Cat. #88518) by wet transfer using Tris-Glycine Transfer Buffer (Bio-Rad, Cat. #1610771) containing 20% methanol at 86 V for the transfer times indicated in Table 1. Precision Plus Protein Standards (Thermo Fisher Scientific, Cat. #BP36031) were included on each gel.

Membranes were blocked with either 3% nonfat dry milk or 3% bovine serum albumin (BSA) in TBST, depending on the target protein, and incubated with primary antibodies overnight at 4 °C or as indicated in Table S1. Membranes were washed three times for 5 min with 0.1% TBST following blocking and after each antibody incubation. HRP-conjugated secondary antibodies were applied for 1 h at room temperature, and immunoreactive proteins were detected using Immobilon Forte Western HRP Substrate (Millipore, Cat. #WBLUF0500). Chemiluminescent images were acquired using an Odyssey Fc Imaging System (LI-COR Biosciences), and molecular weight standards were visualized using the 700 nm fluorescence channel. Protein loading amounts, gel percentages, transfer conditions, blocking buffers, and antibody dilutions for each target are summarized in Table 1.

### 2.8. Bimolecular Fluorescence Complementation (BiFC)

A549/p53-shRNA cells were co-transfected with equimolar amounts of V1-p53 and CerS6-V2 plasmids. Twenty-four hours after transfection, cells were treated with the indicated compounds for either 4 or 24 h before fixation. Reconstitution of Venus fluorescence following p53–CerS6 interaction was visualized by confocal microscopy.

### 2.9. Immunofluorescence and Confocal Microscopy

Cells were seeded in Nunc Lab-Tek II chamber slides (Thermo Fisher Scientific, Cat. #154526), fixed with 4% paraformaldehyde for 10 min at room temperature, permeabilized with 0.1% Triton X-100 in PBS for 10 min, and blocked with 3% BSA in PBS for 1 h. Cells were incubated overnight at 4 °C with anti-calreticulin (CalR; rabbit polyclonal; Invitrogen, Cat. #PA3-900) and/or anti-p53 (mouse monoclonal; Santa Cruz Biotechnology, Dallas, TX, USA; Cat. #sc-126) diluted 1:300 in blocking buffer. Following washes with PBS containing 0.05% Tween-20, cells were incubated for 1 h at room temperature in the dark with the corresponding Alexa Fluor-conjugated secondary antibodies (1:300): donkey anti-rabbit Alexa Fluor 555 (Invitrogen, Cat. #A31572), goat anti-mouse Alexa Fluor 568 (Invitrogen, Cat. #A11031), or chicken anti-mouse Alexa Fluor 647 (Invitrogen, Cat. #A21463). Coverslips were mounted using ProLong Diamond Antifade Mountant with DAPI (Invitrogen, Cat. #P36962). Images were acquired using an Olympus Fluoview FV10i confocal microscope (Olympus Corporation, Hachioji, Tokyo, Japan; 60× objective). Pearson correlation coefficients (Pearson’s R) between Venus BiFC and CalR fluorescence signals were determined using Olympus Fluoview software FV10-ASW 4.2.

### 2.10. Cell Viability Assay

Cell viability was determined using the MTT Cell Proliferation Assay Kit (Abcam, Cambridge, UK; Cat. #ab211091). For hotspot mutant experiments, cells were seeded directly into 96-well plates at 5000 cells per well. For plasmid experiments, cells were transiently transfected in 6-well plates and replated into 96-well plates 24 h later. Cells were allowed to attach overnight before treatment. Cell viability was assessed between 24 and 96 h after treatment, depending on the experiment. MTT reagent (15 μL) was added directly to each well and incubated for 4 h before addition of 100 μL solubilization solution supplied with the kit. Absorbance was measured at 570 nm using a VICTOR™ X5 Multilabel Plate Reader (PerkinElmer, Waltham, MA, USA), with media-only wells used for background correction. Cell viability was expressed as the percentage of the corresponding untreated control for each time point or p53 construct.

### 2.11. Statistical Analysis

Statistical analyses were performed using GraphPad Prism 10 for macOS. Comparisons among multiple groups were performed using one-way analysis of variance (ANOVA) followed by Dunnett’s multiple-comparison test using WT p53 as the reference group. Data are presented as the mean ± SEM. Statistical significance was defined as *p* < 0.05, with significance indicated as: *p* < 0.05 (*), *p* < 0.01 (**), *p* < 0.001 (***), and *p* < 0.0001 (****). Pearson’s correlation coefficients (Pearson’s R) between Venus BiFC and calreticulin (CalR) fluorescence signals were calculated using the Olympus Fluoview FV10i microscope software to quantify their colocalization based on the spatial correspondence and intensity of both signals. Pearson’s R values range from 0 to 1, with values closer to 1 indicating a greater degree of colocalization.

## 3. Results

### 3.1. Structural Requirements for C_16_-Ceramide Binding by p53

**Figure 1:**
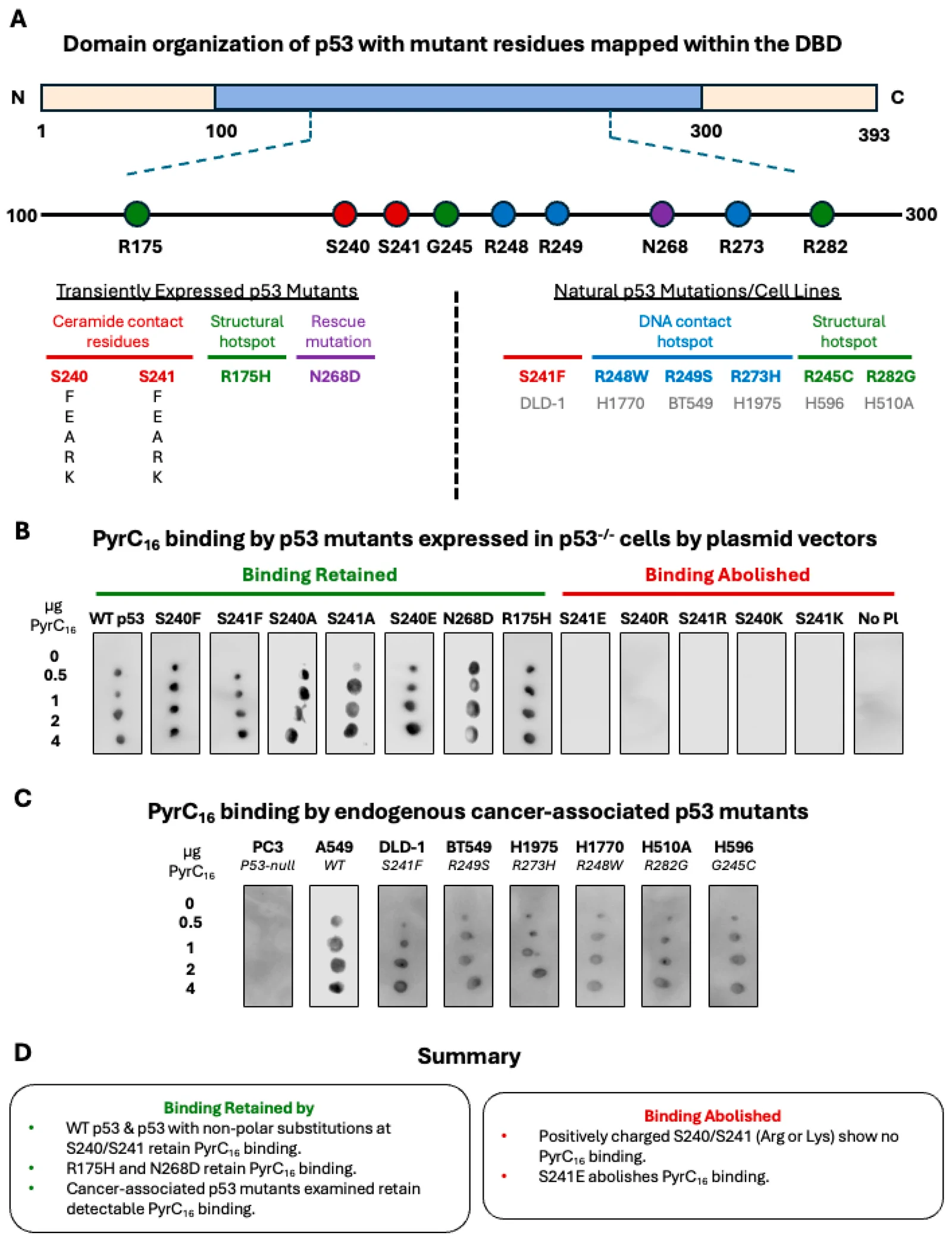
Functional characterization of the p53 ceramide-binding surface. (**A**) The p53 domain organization; DNA-binding domain (DBD) is shown in blue. Inset shows the relative position of DBD residues examined in this study: ceramide contact residues (red); DNA-contact hotspot residues (blue); structural hotspot residues (green); the global suppressor (rescue) mutation N268D (purple). Lists of recombinant p53 mutants (left column) and the natural cancer cell lines-expressed mutants (right column) used in this study are shown. (**B**) Dot blot analysis of PyrC_16_ binding by recombinant p53 mutants. PVDF membranes spotted with increasing amounts of PyrC_16_ (0, 0.5, 1, 2, and 4 μg) were incubated overnight with whole-cell lysates (1 mg/mL) prepared from A549/p53-shRNA cells transiently transfected to express the indicated p53 variants. Bound p53 was detected using an anti-p53 antibody (1:3000) followed by HRP-conjugated secondary antibody (1:5000). Note the differential effects of non-polar and polar substitutions, with positively charged substitutions completely eliminating ceramide binding. WT p53 was used as positive control. All membrane-binding assays were performed in triplicate, and representative images are shown. (**C**) Same analysis as in (**B**) but using lysates obtained from cell lines naturally carrying hot spot p53 mutants. (**D**) Summary of the effects of hot-spot mutations on the interaction between p53 and C_16_-ceramide. Expression of the p53 variants and additional validation of the ceramide-binding assays are shown in Supplemental Figure S1.

Our previous studies demonstrated that residues S240 and S241 serve as C_16_-ceramide contact residues in the p53 DBD [15]. To define the structural and biochemical determinants of this interaction, we generated a panel of amino acid substitutions at both positions designed to evaluate the contributions of residue charge and side-chain identity to ceramide recognition (Figure 1A). The panel included nonpolar (Ala and Phe), negatively charged (Glu), and positively charged (Arg and Lys) substitutions at both residues (Figure 1A). In addition, the structural hotspot mutant R175H and the second-site suppressor mutant N268D, which restores conformational stability to destabilized p53 mutants [21], were examined to determine whether alterations in DBD architecture outside the S240/S241 C_16_-ceramide-binding region affected ceramide binding. Finally, several human cancer cell lines harboring naturally occurring *TP53* mutations (Figure 1A) were analyzed to assess whether clinically relevant mutations retained the ability to bind C_16_-ceramide.

Binding of p53 to ceramide was evaluated using a lipid dot blot assay in which increasing amounts of the water-soluble C_16_-ceramide analog PyrC_16_ were immobilized on PVDF membranes and, after blocking of the membrane, incubated with lysates of cells transfected for expression of the indicated p53 variants (Figure 1B). PyrC_6_, which does not bind p53, served as a negative lipid control (Figure S1C). Since in this experiment p53 variants were transiently expressed in the p53-deficient cell line (exogenous proteins), no treatment was used. Wild-type (WT) p53 exhibited robust binding to PyrC_16_ but not PyrC_6_. Similar binding was observed for S240A, S240F, S240E, S241A, S241F, R175H, and N268D. In contrast, substitution of either S240 or S241 with positively charged residues (Arg or Lys) abolished PyrC_16_ binding. Interestingly, the mutant p53 with negatively charged substitution S241E also failed to bind PyrC_16_, whereas S240E retained binding comparable to WT p53, indicating distinct structural requirements at the two neighboring residues.

To determine whether cancer-associated p53 mutants retained C_16_-ceramide binding, lysates prepared from human cancer cell lines expressing endogenous mutant p53 proteins were analyzed using the same assay (Figure 1C and Figure S1D). PyrC_16_ binding was observed for R248W (NCI-H1770), R249S (BT-549), R273H (NCI-H1975), G245C (NCI-H596), R282G (NCI-H510A), and S241F (DLD-1), whereas no binding was detected in p53-null PC-3 cells. Thus, several common cancer-associated p53 mutants affecting either DNA-contact residues or protein thermodynamic stability retained the ability to bind C_16_-ceramide. As in the experiment with the forced expression of p53 variants (Figure 1B), in this experiment we did not stress cells, which allowed us to assess the p53–PyrC_16_ interaction without potential interference from the endogenous stress-elevated ceramide. Immunoblot analysis confirmed expression of all transfected p53 constructs and endogenous p53 proteins used in the binding assays (Figure S1A,B). Complete dot blot images for all plasmid-derived and endogenous p53 variants are provided in Figure S1C,D.

While these experiments did not provide quantitative characterization of the interactions studied, they allowed us to separate p53 mutants which would be unlikely to support signaling by ceramide. Moreover, the obtained results defined the structural requirements for C_16_-ceramide recognition by p53. Specifically, the introduction of a positive charge at either S240 or S241 consistently disrupted ceramide binding, whereas multiple substitutions at neighboring residues and several common cancer-associated mutations retained binding activity. These findings indicate that C_16_-ceramide recognition is governed primarily by the local biochemical environment of the S240/S241 region and can be maintained despite diverse structural perturbations within the DBD (Figure 1D).

### 3.2. C_16_-Ceramide Binding Is Associated with Stress-Induced Interaction Between p53 and CerS6

**Figure 2:**
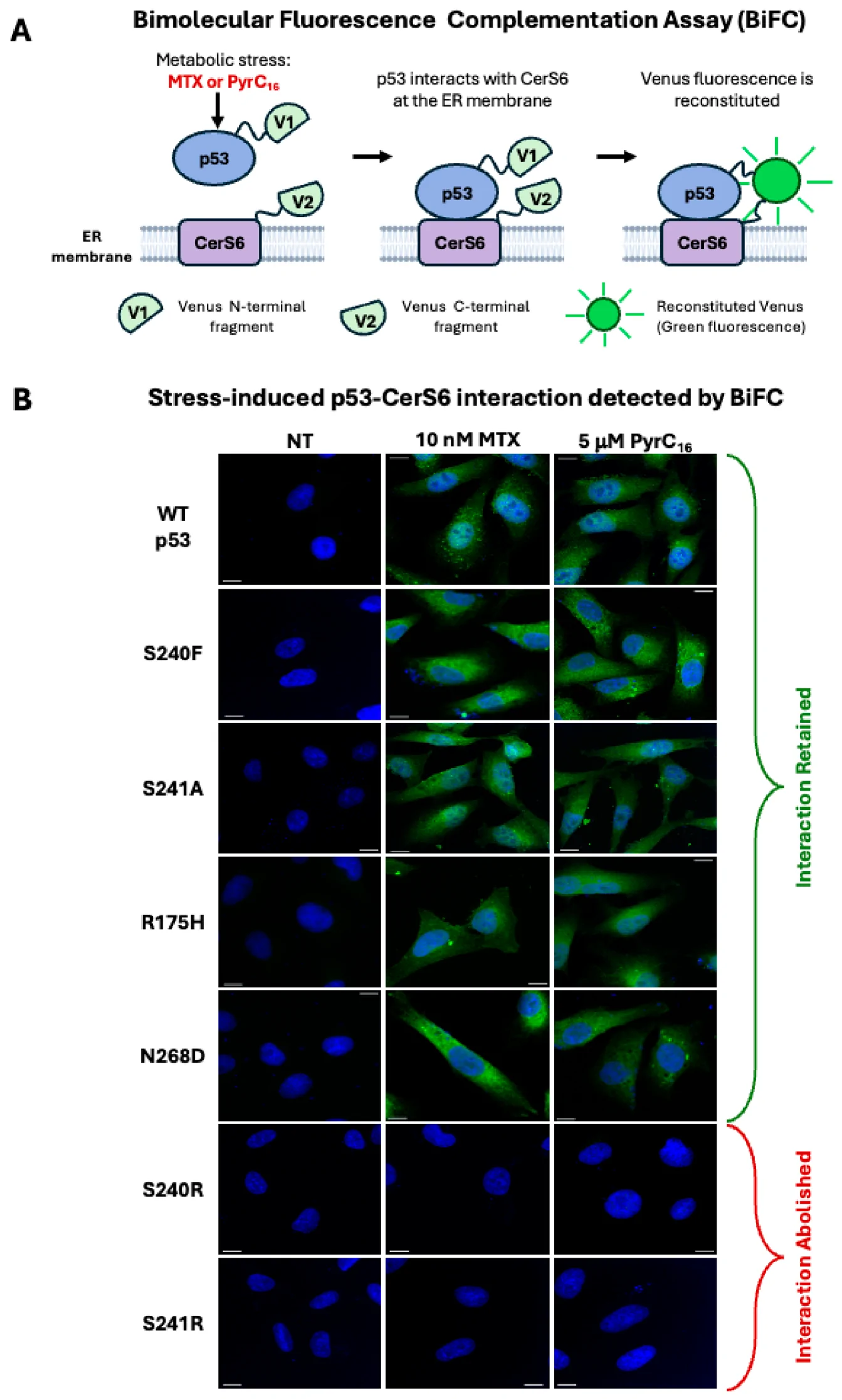
Effect of p53 mutations on the p53–CerS6 interaction. (**A**) Schematic illustrating the bimolecular fluorescence complementation (BiFC) assay used to detect p53–CerS6 interaction. The N-terminal Venus fragment (V1) was fused to the N-terminus of p53 (V1-p53) and the C-terminal Venus fragment (V2) was fused to the C-terminus of CerS6 (CerS6-V2). The Venus fragments are non-fluorescing on their own. When the fused proteins, p53 and CerS6, interact with each other, the Venus fragments are brought into proximity of each other, resulting in Venus reconstitution and green fluorescence. (**B**) Non-stressed (NT) cells transfected with BiFC vectors pair do not exhibit fluorescence (no interaction). Metabolic stress induced by MTX or PyrC_16_ promotes interaction between V1-p53 and CerS6-V2, resulting in reconstitution of Venus fluorescence at the endoplasmic reticulum (ER). Representative BiFC images of A549/p53-shRNA cells co-expressing V1-p53 variants and CerS6-V2, following no treatment (NT), or treated with MTX (10 nM, 24 h), or PyrC_16_ (5 μM, 24 h). BiFC fluorescence (green) indicates p53–CerS6 interaction; nuclei were counterstained with DAPI (blue). Images were acquired using an Olympus Fluoview FV10i confocal microscope at 140× magnification. Scale bars, 20 μm. BiFC assays were performed in triplicates, and representative images are shown. 100% of cells were transfected with BiFC plasmids and showed complementation (green) upon stress. BiFC co-localization with calreticulin (CalR) on ER is shown in Figure S2.

Previous studies demonstrated that metabolic stress promotes p53 interaction with CerS6, the ER-resident enzyme responsible for C_16_-ceramide synthesis, through the p53 DBD [18]. To investigate how mutations in the ceramide-accommodating region of the p53 DBD affect stress-induced p53–CerS6 complex formation, select p53 variants that retained or lost PyrC_16_ binding were analyzed by bimolecular fluorescence complementation (BiFC), an approach that allows direct monitoring of protein–protein interactions (Figure 2A) [28]. In agreement with our previous reports [30], WT p53 produced little or no detectable BiFC fluorescence in unstressed cells, indicating the absence of detectable interaction between the two proteins in the absence of metabolic stress (Figure 2B). In contrast, robust fluorescence was observed following treatment with either methotrexate (MTX) or PyrC_16_ (Figure 2B). Similar stress-induced BiFC was observed for S240F, S241A, R175H, and N268D p53 mutants, all of which retained PyrC_16_ binding (Figure 1B and Figure 2B). S240R and S241R mutants, which failed to bind PyrC_16_, did not produce detectable BiFC following treatment with either compound (Figure 2B). To verify the subcellular localization of the interaction, cells were co-stained with the ER marker calreticulin. In all interaction-positive variants, the reconstituted Venus signal co-localized with calreticulin, confirming that the stress-induced p53–CerS6 interaction occurred at the ER membrane (Figure S2). Analysis of the complete S240/S241 substitution panel (Figure S2A–G) demonstrated the same relationship between PyrC_16_ binding and BiFC as was observed for the representative mutants shown in Figure 2B. Immunoblot analysis confirmed comparable expression of all BiFC constructs (Figure S2H).

Together, these findings indicate that stress-induced p53–CerS6 interaction is dependent on C_16_-ceramide-binding competence. This is in agreement with our latest study, which demonstrated that the ability of p53 to bind ceramide is essential for the p53–CerS6 interaction on the ER membrane [18]. Consistent with such relationship, mutations that abolished PyrC_16_ binding uniformly failed to produce stress-induced BiFC, whereas ceramide-binding-competent mutants retained stress-induced interaction with CerS6.

### 3.3. Disruption of C_16_-Ceramide Binding Attenuates Activation of the p53 Pathway During Metabolic Stress

**Figure 3:**
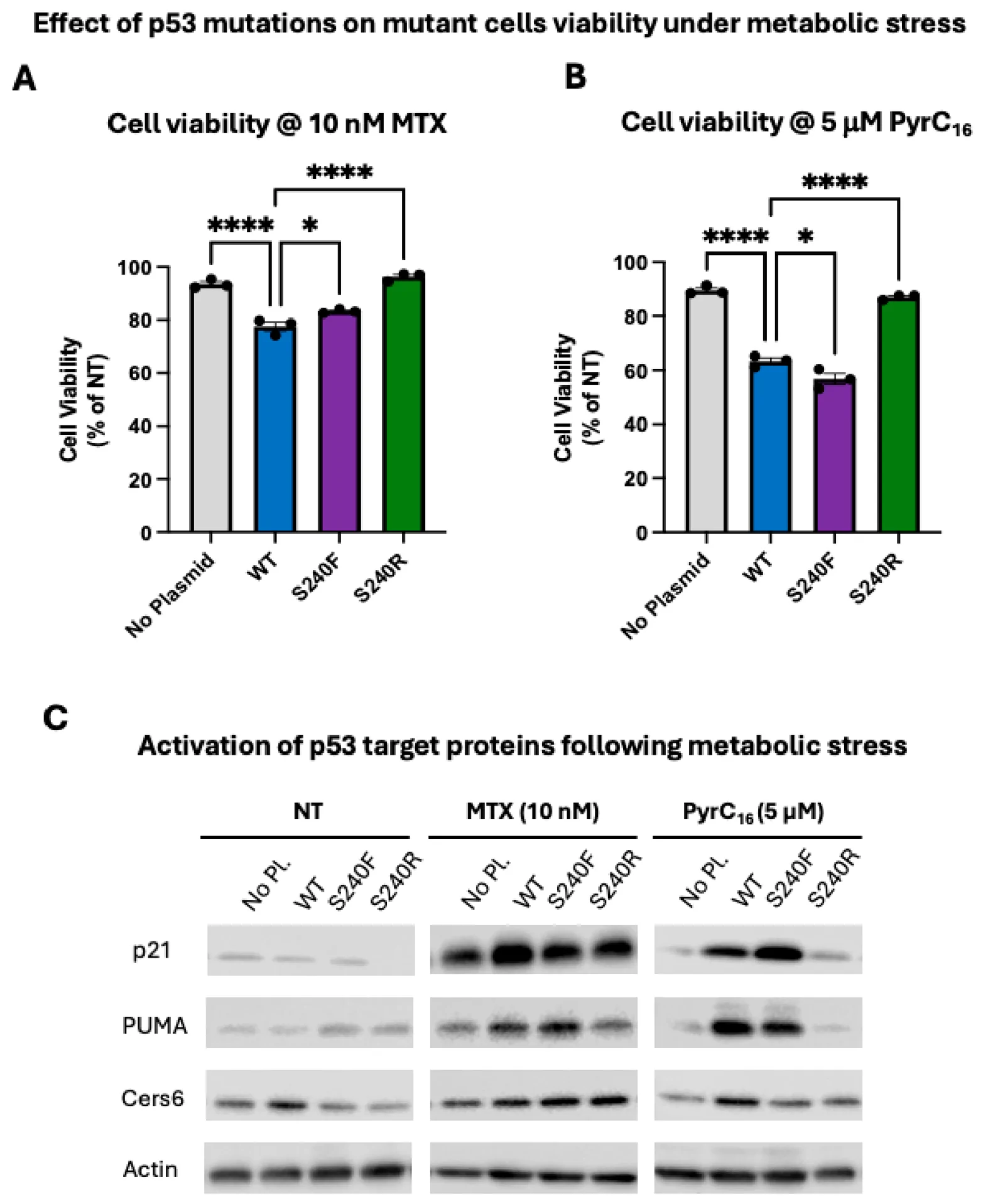
Functional consequences of disrupting the p53 ceramide-binding surface. Viability of A549/p53-shRNA cells transiently expressing WT or mutant (S240F or S240R) p53 and treated for 24 h with (**A**) MTX (10 nM) or (**B**) PyrC_16_ (5 μM). Cell viability was determined by MTT assay and normalized to the corresponding NT control for each plasmid. Data are presented as mean ± SEM. Statistical significance was determined by one-way ANOVA with Dunnett’s multiple-comparison test. * *p* < 0.05; **** *p* < 0.0001. Data represent mean ± SEM from three independent experiments (n = 3), each performed with six technical replicates per condition. Cancer cells expressing PyrC_16_-binding-proficient p53 mutants are sensitive to both classes of drugs, whereas cells expressing PyrC_16_-binding-deficient p53 mutants are not responsive to either drug class. (**C**) Immunoblot analysis (whole-cell lysates) of p53 downstream targets in A549/p53-shRNA cells treated as in (**A**,**B**) demonstrates activation of downstream targets only in cells expressing PyrC_16_-binding-proficient p53 mutants. Actin served as the loading control. Representative blots from three experiments are shown.

Having established that C_16_-ceramide binding promotes stress-induced interaction between p53 and CerS6 (Figure 1 and Figure 2), we next asked whether disruption of this pathway alters downstream p53 signaling during metabolic stress. To test this, A549/p53-shRNA cells were transiently transfected with WT p53 or two representative mutants: S240F, which retains both C_16_-ceramide binding and stress-induced interaction with CerS6, and S240R, which lacks the ability to bind ceramide or interact with CerS6. Treatment with either MTX or PyrC_16_ significantly reduced viability of WT p53-expressing cells compared to cells lacking p53 (A549/p53-shRNA) (Figure 3A,B). Similar reduction in viability for both MTX and PyrC_16_ was observed in cells expressing the ceramide-binding-competent S240F mutant. In contrast, cells expressing the ceramide-binding-deficient S240R mutant showed weaker responses to drug treatments, which were at the level of p53-deficient cells (Figure 3A,B), indicating reduced sensitivity to drug-induced metabolic stress. Time-course analysis further demonstrated that the responses of the ceramide-binding-competent mutants R175H and N268D closely paralleled those of WT p53 and S240F, whereas S240R consistently displayed the greatest resistance to both MTX- and PyrC_16_-induced growth inhibition (Figure S3A). Consistent with the p53-dependent response to MTX and PyrC_16_, increased expression of the p53 downstream targets p21, PUMA, and CerS6 was seen in cells expressing WT p53 or the S240F mutant (Figure 3C). In contrast, expression of all three target proteins was attenuated in cells expressing the S240R mutant (Figure 3C; complete immunoblots are shown in Figure S3B). This effect was especially profound for canonical p53 targets, p21 and PUMA. While MTX showed a similar trend (Figure 3C), the outcomes were not as clear as in the case of PyrC_16_. We interpret this as the result of potentially pleiotropic effects of MTX, which also explains the activation of p21 in the absence of p53. Further, it is expected that through direct binding, PyrC_16_ activates the p53 responses more rapidly than MTX, the effect of which on p53 is indirect.

Because CerS6 is both a transcriptional target of p53 and the ER-resident enzyme responsible for C_16_-ceramide synthesis, reduced CerS6 induction by ceramide-binding-deficient p53 mutants is consistent with diminished activation of the CerS6–p53 feed-forward loop [15]. Together, these findings suggest that loss of C_16_-ceramide binding is associated with attenuated activation of the p53 pathway and reduced cellular sensitivity to MTX and PyrC_16_.

### 3.4. Cancer-Associated p53 Mutants Exhibit Differential Responses to Metabolic Stress

**Figure 4:**
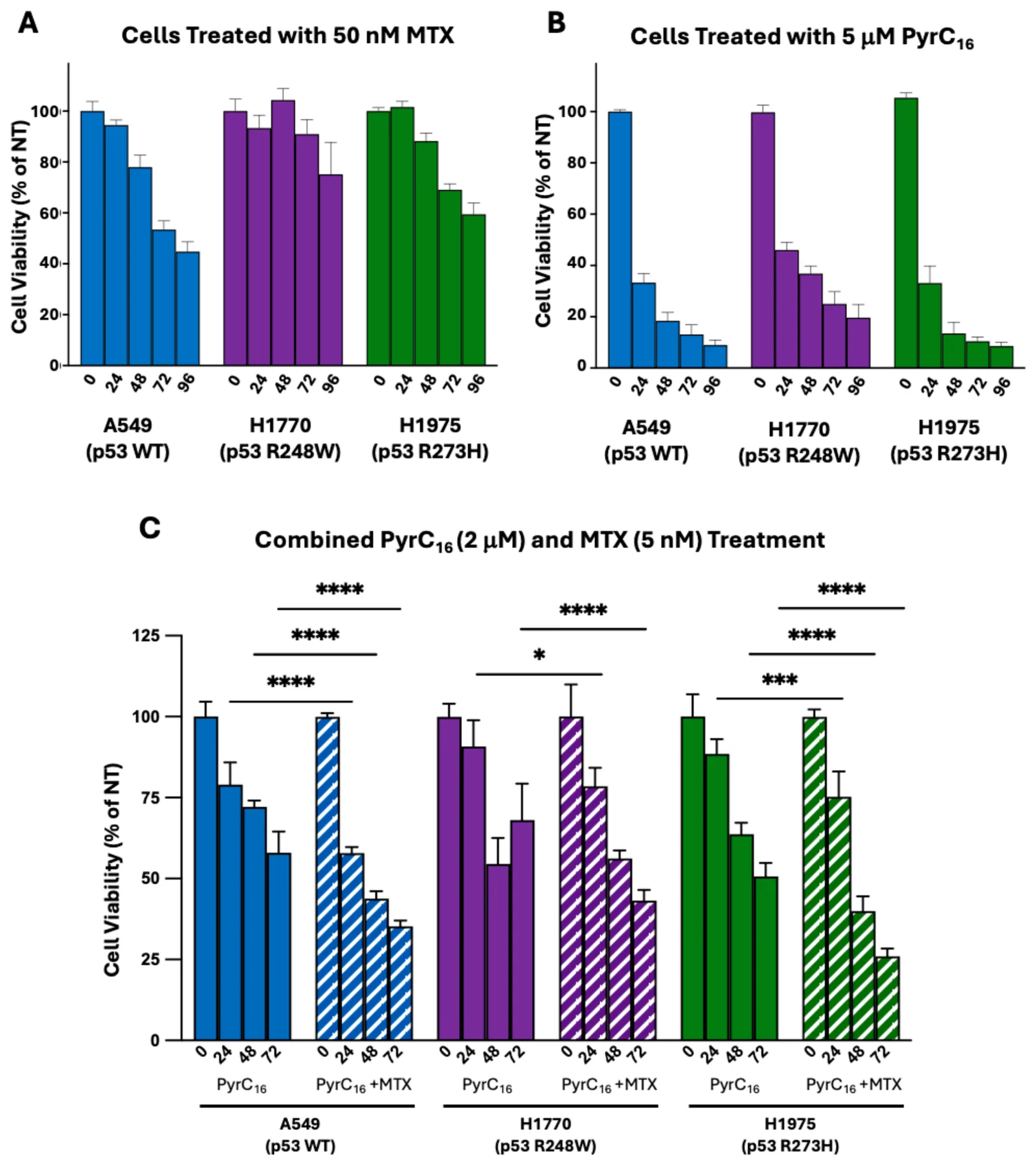
Time-dependent responses to drug treatments in cell lines carrying different cancer-associated p53 mutants. (**A**) MTT analysis of cell viability in A549 (WT p53), NCI-H1770 (R248W), and NCI-H1975 (R273H) cells following continuous treatment with MTX (50 nM). Cell viability was measured after 0, 24, 48, and 72 h of treatment and normalized to the corresponding NT control for each cell line. Blue, A549 cells; purple, H1770 cells; green, H1975 cells. Numbers on *X*-axis show hours of treatment; replicate number for all cell lines and treatment is n = 6. Data are presented as mean ± SEM. (**B**) MTT analysis of cell viability in A549 (WT p53), NCI-H1770 (R248W), and NCI-H1975 (R273H) cells following continuous treatment with PyrC_16_ (5 μM). Cell viability was measured after 0, 24, 48, and 72 h of treatment and normalized to the corresponding NT control for each cell line. Data are presented as mean ± SEM. (**C**) MTT analysis of cell viability following combined treatment with the sub-optimal doses of the above drugs (MTX at 10% and PyrC_16_ at 40%). A549 (WT p53), NCI-H1770 (R248W), and NCI-H1975 (R273H) cells were treated continuously with PyrC_16_ (2 μM) alone or in combination with MTX (5 nM), and cell viability was determined after 0, 24, 48, and 72 h. Cell viability was normalized to the corresponding NT control for each cell line. Data are presented as mean ± SEM. Blue, A549 cells; purple, H1770 cells; green, H1975 cells; hatched bars correspond to the same cells treated with the combination of drugs. Numbers on *X*-axis show hours of treatment; replicate number for all cell lines and treatments is n = 6. The drug combination produced a greater reduction in cell viability than either agent alone, suggesting that PyrC_16_ potentiates the effects of MTX. Statistical analysis of effect differences between single-drug treatment and the combination of PyrC_16_ with MTX were performed using two-way ANOVA with Sidak’s multiple comparisons test. * *p* < 0.05; *** *p* < 0.001, **** *p* < 0.0001.

To determine whether the relationship between C_16_-ceramide binding and cellular responses to metabolic stress extended to endogenous cancer-associated p53 mutants, A549 cells expressing WT p53 and the representative hotspot mutant cell lines NCI-H1770 (R248W) and NCI-H1975 (R273H), both of which retained C_16_-ceramide binding (Figure 1C), were treated with MTX, PyrC_16_, or the combination of both agents, and cell viability was assessed by MTT assay (Figure 4). R248W and R273H, representative DNA-contact hotspot mutants, were selected because both retained C_16_-ceramide binding, allowing us to determine whether C_16_-ceramide-dependent signaling was preserved in clinically relevant *TP53* mutants. Treatment with MTX alone produced a time-dependent reduction in cell viability in all three cell lines, although the magnitude of the response differed between mutants (Figure 4A). In contrast, PyrC_16_ produced a substantially greater reduction in viability than MTX alone, with all three cell lines exhibiting marked sensitivity to PyrC_16_ (Figure 4B). While the kinetics of the response varied, both endogenous p53 mutants retained sensitivity to direct C_16_-ceramide exposure.

To determine whether direct C_16_-ceramide delivery could modify the response to metabolic stress under submaximal conditions, cells were treated with sub-IC_50_ concentrations of MTX and PyrC_16_ in combination (Figure 4C). Lower concentrations were selected because 5 μM PyrC_16_ alone produced substantial loss of viability, limiting the ability to detect additional effects of combination treatment. Under these conditions, combined treatment consistently reduced cell viability, indicating that exogenous C_16_-ceramide potentiated the cellular response to metabolic stress-induced growth inhibition. Similar trends were observed across the complete panel of endogenous p53 mutant cell lines, including R249S (BT-549), G245C (NCI-H596), and S241F (DLD-1) (Figure S4A).

Because these experiments compared multiple human cancer cell lines with distinct genetic backgrounds, endogenous expression of p53 and CerS6 was confirmed by immunoblot analysis (Figure S4B). Together, these findings demonstrate that endogenous cancer-associated p53 mutants retaining C_16_-ceramide binding remain capable of responding to metabolic stress, although the magnitude and kinetics of these effects vary among individual *TP53* mutants.

### 3.5. Metabolic Stress-Induced p53–CerS6 Complex Formation and ER Remodeling Occur Independently of Canonical UPR Activation

Our previous studies revealed that metabolic stress induced formation of large p53–CerS6 membrane assemblies at the endoplasmic reticulum (ER) [30]. These reversible structures resemble ER remodeling events observed during an evolutionarily conserved form of stress adaptation that is unrelated to the unfolded protein response (UPR) [31,32]. To determine whether the structures represent a distinct stress response, we investigated whether the canonical unfolded protein response (UPR) was associated with p53–CerS6 complex formation.

**Figure 5:**
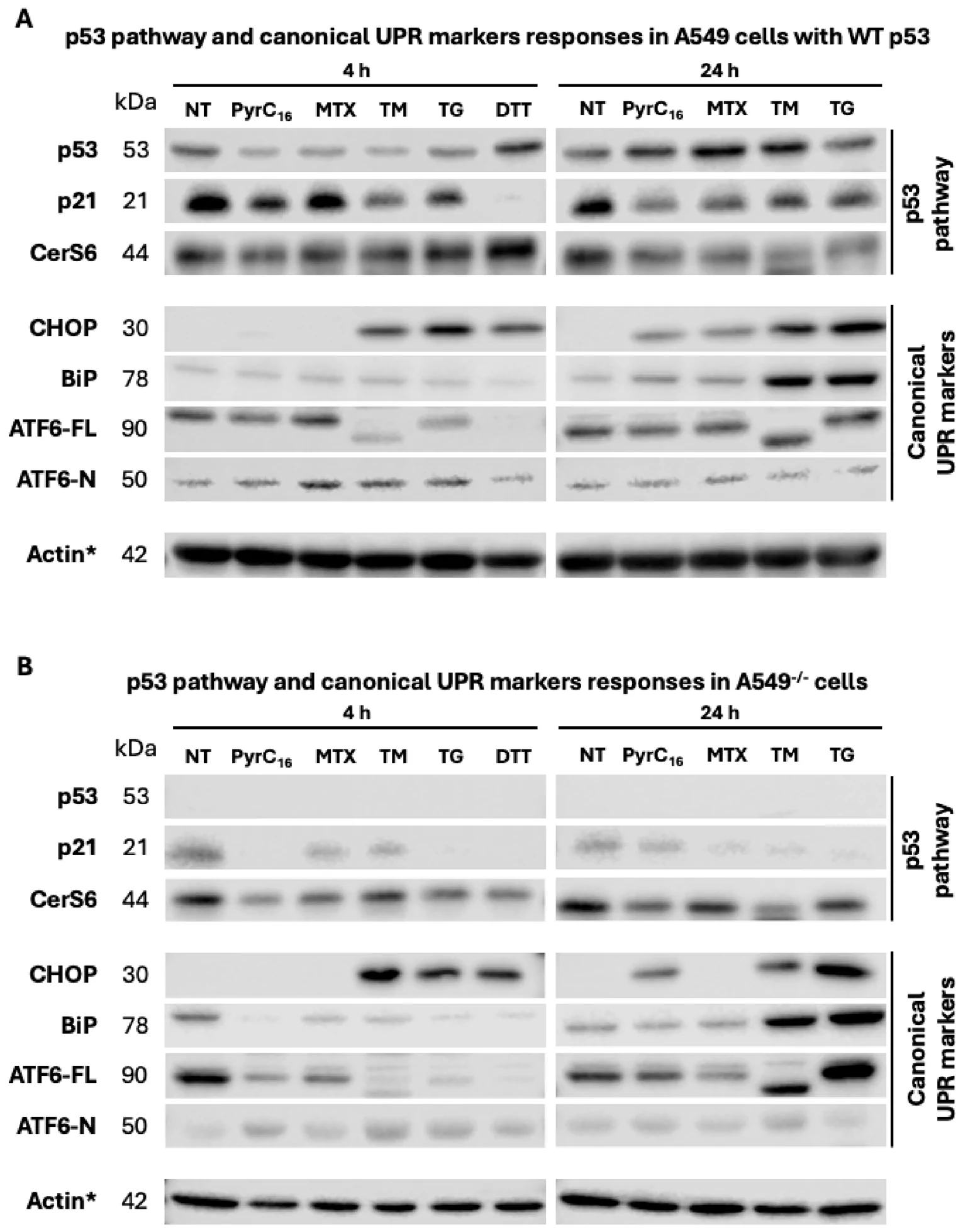

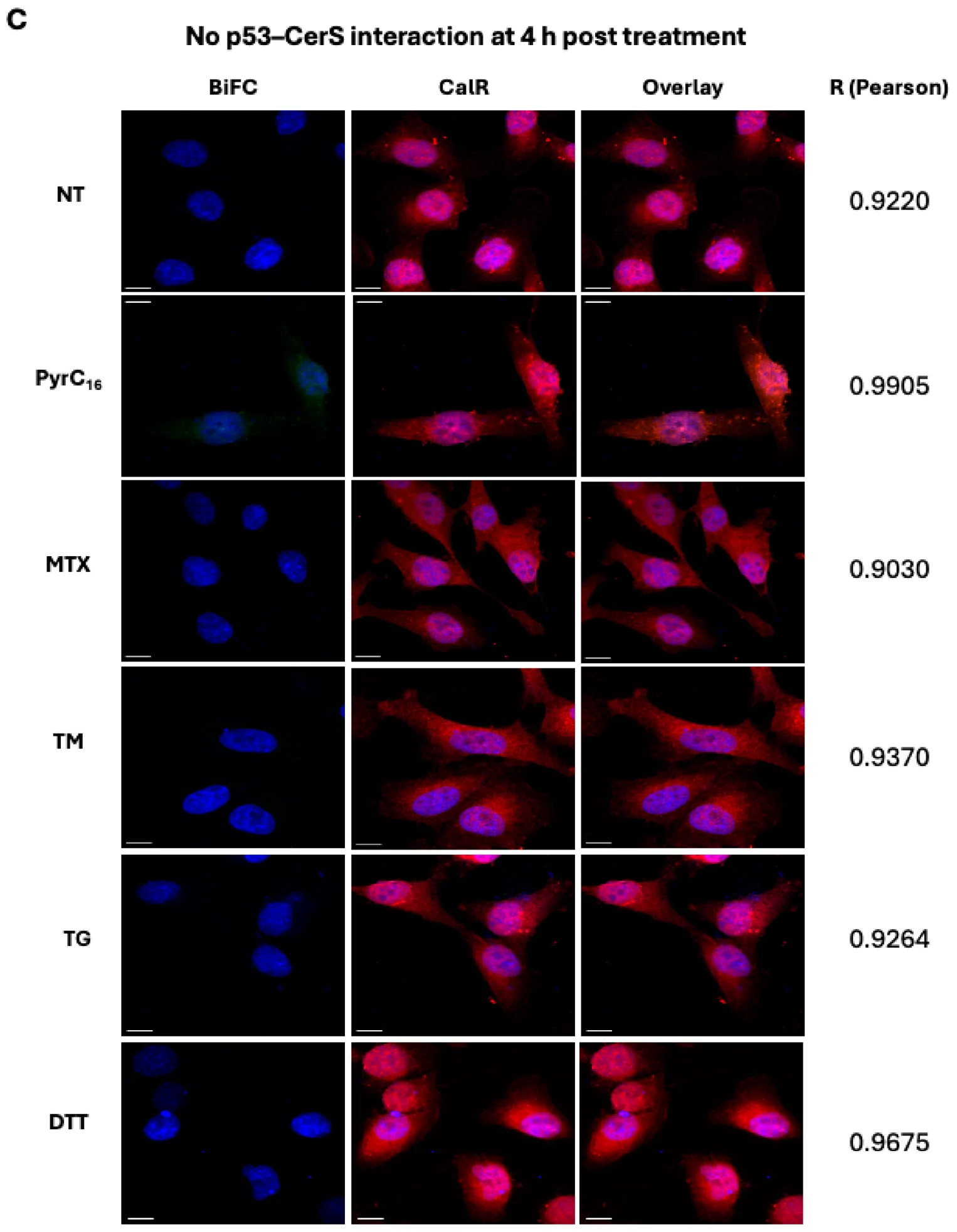

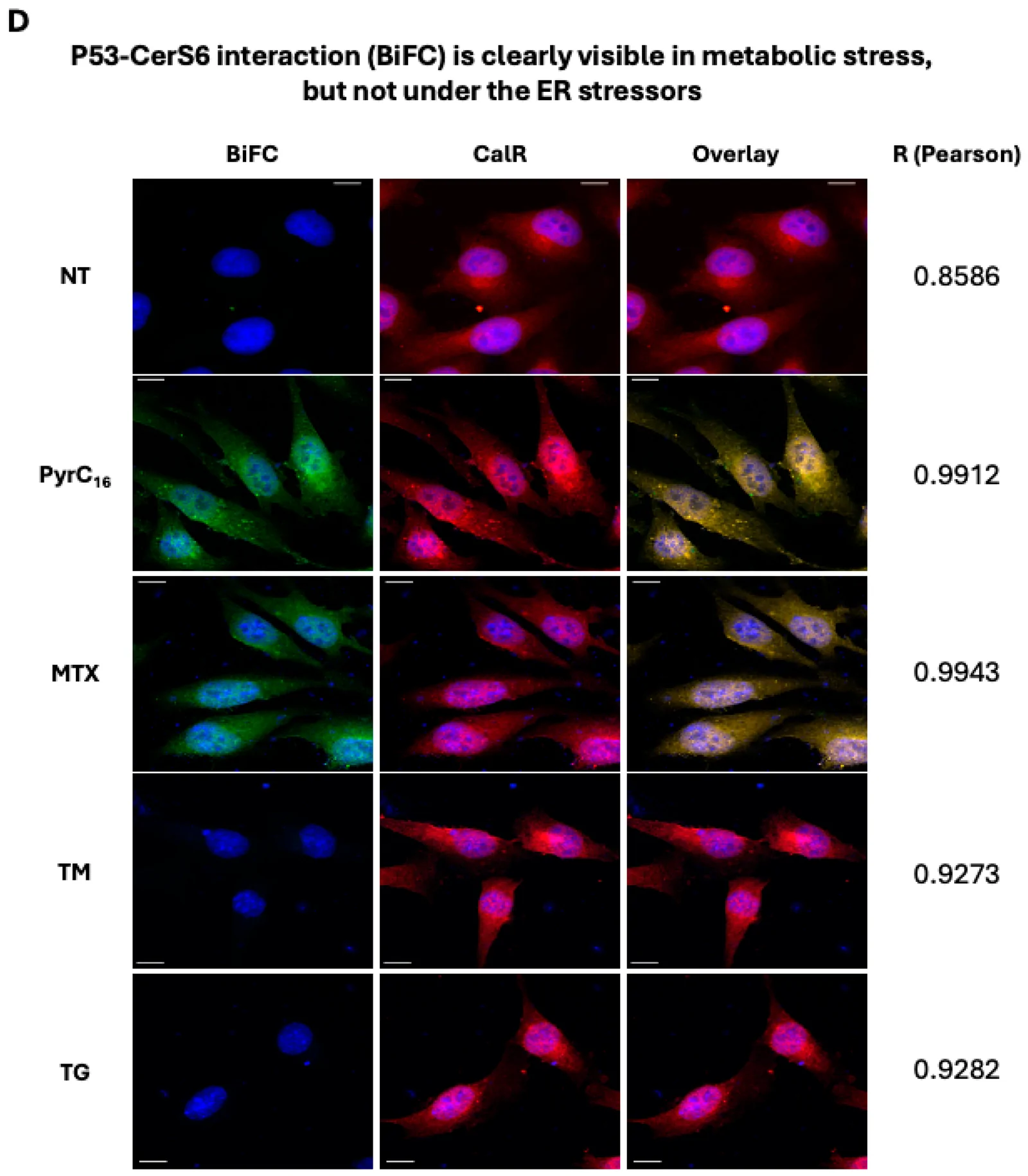
Drugs inducing metabolic stress promote p53–CerS6 interaction on the ER and ceramide signaling response independently of canonical UPR. (**A**) Immunoblot analysis of p53 pathway proteins and canonical unfolded protein response (UPR) markers in A549 WT cells following treatment with metabolic or ER stress-inducing agents. Cells were treated with MTX (100 nM, 4 h; 10 nM, 24 h), PyrC_16_ (5 μM, 4 and 24 h), tunicamycin (TM; 10 μg/mL, 4 h; 4 μg/mL, 24 h), thapsigargin (TG; 1 μM, 4 h; 0.2 μM, 24 h), or dithiothreitol (DTT; 4 mM, 4 h). Whole-cell lysates were analyzed by immunoblotting for p53, p21, CerS6, CHOP, BiP, full-length ATF6 (ATF6-FL), and cleaved ATF6 (ATF6-N). Representative actin loading controls are shown (Actin*); corresponding loading controls for each immunoblot are presented in Figure S5. (**B**) Immunoblot analysis of p53 pathway proteins and canonical UPR markers in A549/p53-shRNA cells treated under the same conditions as in (**A**). Whole-cell lysates were analyzed for the indicated proteins to distinguish p53-dependent responses from canonical UPR activation. (**C**) Representative BiFC images of A549/p53-shRNA cells co-expressing V1-p53 and CerS6-V2 following 4 h treatment with the indicated agents. BiFC fluorescence (green) is missing, CalR immunofluorescence (red) labels the endoplasmic reticulum, nuclei were counterstained with DAPI (blue). Merged images show only nuclear and ER signals, no colocalization. Pearson’s correlation coefficients (R) are shown for each condition. Scale bars, 20 μm. 140× magnification. No BiFC was observed at the 4 h time point. (**D**) Representative BiFC images of A549/p53-shRNA cells co-expressing V1-p53 and CerS6-V2 following 24 h treatment with the indicated agents. BiFC fluorescence (green) indicates p53–CerS6 interaction, CalR immunofluorescence (red) labels the endoplasmic reticulum, nuclei were counterstained with DAPI (blue), and merged images (yellow) indicate co-localization of the BiFC signal with the ER. BiFC assays were performed in triplicates, and representative images are shown. Pearson’s correlation coefficients (R) are shown for each condition. Scale bars, 20 μm. 140× magnification. Prominent p53–CerS6 interaction was observed at the 24 h time point only following treatment for the metabolic stressors tested. No p53–CerS6 interaction was observed upon induction of UPR.

WT and p53-null A549 cells were treated with the metabolic stressors MTX and PyrC_16_ or with the established UPR inducers tunicamycin (TM), thapsigargin (TG), and dithiothreitol (DTT). These agents activate the canonical UPR through distinct mechanisms, allowing metabolic stress-specific responses to be distinguished from generalized ER stress. Activation of the p53 pathway and canonical UPR markers was evaluated by immunoblot assays at mid- (4 h) and late- (24 h) phases of time response to determine whether p53–CerS6 complex formation preceded, coincided with, or occurred independently of canonical UPR activation (Figure 5A,B). DTT was evaluated only at the mid- time point because prolonged treatment markedly reduced cell viability. Early time points were not investigated for UPR markers, because our previous data indicated that p53 localization to the ER can be noted at about 2 h post-stress induction [18].

At 4 h, TM, TG, and DTT induced canonical UPR signaling, as evidenced by increased CHOP and BiP expression along with ATF6 processing. This response remained evident or became more pronounced after 24 h for TM and TG. In contrast, MTX and PyrC_16_ increased expression of p53, p21, and CerS6 while producing little or no activation of canonical UPR markers at either time point. As expected, induction of the p53 pathway was absent in A549/p53-shRNA cells, whereas activation of canonical UPR markers by TM, TG, and DTT was preserved, confirming that these pathways are activated independently of p53. Complete immunoblots, lane assignments, and molecular weight references are provided in Figure S5.

BiFC analysis was next performed to determine whether activation of the canonical UPR was sufficient to promote p53–CerS6 interaction (Figure 5C,D). At the early 4 h time point, TM, TG, and DTT had already induced robust UPR signaling; however, none of these treatments generated detectable BiFC, even at 24 h. Likewise, MTX produced no detectable BiFC at 4 h, whereas PyrC_16_ produced only weak fluorescence, consistent with the earlier onset of signaling following direct C_16_-ceramide delivery. By 24 h, both MTX- and PyrC_16_-treated cells exhibited robust BiFC that co-localized with the ER marker calreticulin, whereas TM and TG remained BiFC-negative despite sustained activation of the canonical UPR. Pearson correlation analysis confirmed strong colocalization of the BiFC signal with calreticulin under conditions in which interaction occurred.

Together, these findings demonstrate that p53–CerS6 complex formation occurs independently of canonical UPR activation and is not a general consequence of ER stress.

### 3.6. Formation of the p53–CerS6 Complex Is Induced by a Selective Subset of p53-Activating Stimuli

**Figure 6:**
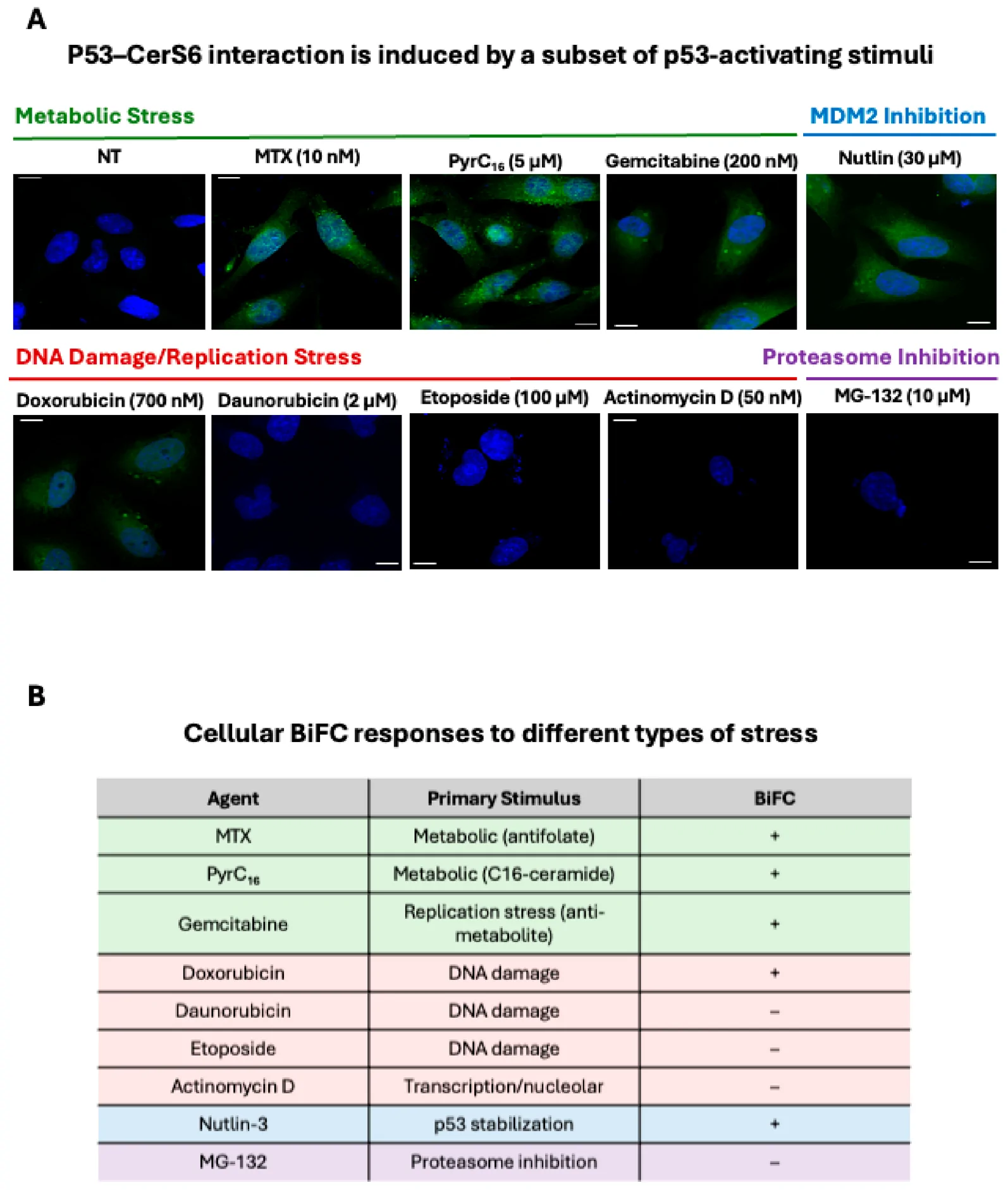
Selective induction of p53–CerS6 interaction by distinct cellular stressors. (**A**) Representative BiFC images of A549/p53-shRNA cells transiently co-expressing V1-p53 and CerS6-V2 following 24 h treatment with the indicated agents. Cells were treated with MTX (10 nM), PyrC_16_ (5 μM), gemcitabine (200 nM), Nutlin-3 (30 μM), doxorubicin (700 nM), daunorubicin (2 μM), etoposide (100 μM), actinomycin D (50 nM), or MG-132 (10 μM). BiFC fluorescence (green) indicates p53–CerS6 interaction; nuclei were counterstained with DAPI (blue). All BiFC assays were performed in triplicates, and representative images are shown. CalR co-localization and Pearson’s correlation analysis are presented in Figure S6. Scale bars, 20 μm. 140× magnification. (**B**) Summary of the primary cellular stimuli represented by each treatment and the corresponding p53–CerS6 BiFC response. “+” indicates detectable BiFC and “−” indicates no detectable BiFC under the conditions tested. Table colors indicate the types of stressors used for BiFC.

Having established that formation of the p53–CerS6 complex is independent of canonical UPR activation, we next examined whether the interaction represents a universal consequence of p53 activation. Previously, we demonstrated that, unlike MTX, the antifolate lometrexol failed to induce detectable p53–CerS6 interaction despite eliciting metabolic stress [30]. We therefore expanded our analysis to include additional stimuli that activate p53 through distinct mechanisms, including metabolic stress (MTX and PyrC_16_), replication stress (gemcitabine), DNA damage (doxorubicin, daunorubicin, etoposide, and actinomycin D), MDM2 inhibition (Nutlin-3), and proteasome inhibition (MG-132) (Figure 6). Consistent with previous observations, MTX and PyrC_16_ induced robust BiFC. Gemcitabine likewise led to formation of the p53–CerS6 complex, demonstrating that recruitment of p53 to CerS6 is not restricted to antifolate treatment. Among the DNA-damaging agents examined, doxorubicin also produced detectable BiFC, whereas daunorubicin, etoposide, and actinomycin D failed to induce interaction under the conditions tested. Nutlin-3 induced robust BiFC, whereas the proteasome inhibitor MG-132 failed to produce detectable interaction despite p53 accumulation resulting from inhibition of proteasomal degradation.

Together with the previous observation that lometrexol does not induce p53–CerS6 interaction, these findings demonstrate that formation of the p53–CerS6 complex is not a universal consequence of p53 activation. Rather, recruitment of p53 to CerS6 occurs only in response to a selective subset of cellular stimuli, indicating that activation of the CerS6–p53 signaling pathway is stimulus dependent.

## 4. Discussion

The tumor suppressor p53 integrates a wide range of cellular stress signals to coordinate cell-cycle arrest, apoptosis, and metabolic adaptation [33,34]. Although metabolic stress is well recognized as a potent activator of p53 [33,34], the molecular mechanisms by which changes in lipid metabolism are communicated to the p53 signaling network remain poorly understood. Here, we characterize a previously unknown lipid-recognition surface within the p53 DBD centered on residues S240 and S241 and demonstrate that direct C_16_-ceramide binding is required for stress-induced assembly of the CerS6–p53 signaling complex and efficient activation of downstream p53 pathways. Importantly, this ceramide-binding activity is retained by several common cancer-associated p53 mutants, indicating that lipid recognition is mechanistically distinct from the DNA-binding defects characteristic of many tumor-derived p53 variants. When considered together with our previous studies demonstrating that CerS6-derived C_16_-ceramide thermodynamically stabilizes p53 and prevents MDM2-mediated p53 degradation [15], these findings support a model in which CerS6 functions not only as the source of a bioactive lipid but also as a platform that directly couples metabolic stress to p53 activation through localized C_16_-ceramide signaling at the endoplasmic reticulum.

A central finding of the present study is that C_16_-ceramide recognition depends on the biochemical properties of a discrete regulatory surface within the p53 DBD. Systematic mutational analysis demonstrated that C_16_-ceramide recognition is governed by distinct structural requirements at residues S240 and S241 rather than simple permissiveness for lipid binding. Introduction of positively charged residues at either position consistently abolished ceramide binding, stress-induced interaction with CerS6, and downstream activation of the p53 pathway, whereas substitutions with alanine or phenylalanine were both well tolerated. The differential effects of the glutamate substitutions further indicate that S240 and S241 make non-equivalent contributions to ligand recognition despite their close proximity. Together, these findings establish S240/S241 as a bona fide lipid-recognition surface whose local biochemical environment, rather than the overall structural integrity of the DBD, governs C_16_-ceramide binding.

Beyond identifying the ceramide-binding surface, one of the most notable findings of this study is its localization within the p53 global suppressor region. Residue S240 lies within a structural motif in which second-site suppressor mutations restore stability and function to a broad spectrum of destabilized p53 mutants by promoting the native (non-mutant) conformation of the DBD [21,35]. Although the present study was not designed to examine the mechanisms underlying global suppression, the observation that the suppressor mutant N268D retained C_16_-ceramide binding and stress-induced interaction with CerS6 suggests that lipid recognition is compatible with structural stabilization mediated through this region. Consistent with this interpretation, previous studies demonstrated that C_16_-ceramide binding increases the thermal stability of the isolated p53 DNA-binding domain [15]. Taken together, these findings identify the ceramide-binding surface as a previously unrecognized regulatory interface within the p53 global suppressor region. Whether C_16_-ceramide binding and global suppressor mutations influence p53 stability through related structural mechanisms remains an important question for future investigation. Further structural studies will be required to determine how C_16_-ceramide binding influences the conformational dynamics of this regulatory surface.

Beyond defining the structural basis of C_16_-ceramide recognition, the present study demonstrates that lipid binding is functionally required for efficient activation of the CerS6–p53 signaling pathway. Mutations that disrupted C_16_-ceramide binding uniformly prevented stress-induced interaction between p53 and CerS6 and attenuated induction of the downstream p53 targets p21, PUMA, and CerS6 during metabolic stress. Conversely, the examined lipid-binding p53 mutants retained both CerS6 interaction and downstream signaling, indicating that C_16_-ceramide recognition is an essential upstream event in pathway activation rather than simply a consequence of p53 stabilization. Of note, we cannot completely exclude additional effects of the mutations on p53 function beyond ceramide binding, including their effects on the local folding, DNA binding or interactions with co-activators. Nevertheless, these findings extend the previous model of C_16_-ceramide-mediated p53 stabilization by demonstrating that direct lipid recognition is also required for efficient stress-induced interaction between p53 and CerS6 and activation of downstream p53 signaling [15]. Together, these observations support a feed-forward mechanism in which metabolic stress promotes localized generation of C_16_-ceramide by CerS6, stress-induced interaction between p53 and CerS6 at the endoplasmic reticulum, direct channeling of C_16_-ceramide to p53 [18,30], stabilization and activation of the protein, and transcriptional induction of CerS6 to amplify the cellular response.

An additional important finding of this study is that several clinically relevant *TP53* hotspot mutants retained C_16_-ceramide binding and responsiveness to metabolic stress. These mutations disrupt p53 function through diverse mechanisms, including impaired DNA binding and structural destabilization [36,37], indicating that the ceramide-binding surface remains functional despite substantial alterations elsewhere in the DBD. Moreover, combined treatment with MTX and PyrC_16_ produced greater reductions in cell viability than either agent alone in multiple mutant cell lines, suggesting that direct C_16_-ceramide delivery can enhance cellular responses to metabolic stress even in the presence of common *TP53* mutations. Together, these findings indicate that the ceramide-binding surface is structurally and functionally separable from many of the canonical interfaces disrupted due to tumorigenesis. Whether retained C_16_-ceramide binding contributes to residual p53 activity in specific mutant backgrounds or could potentially be exploited therapeutically to enhance mutant p53 function remains an intriguing question for future investigation.

The present study also provides new insight into the nature of the stress-induced p53–CerS6 structures observed at the endoplasmic reticulum. Because these reversible structures resemble ER remodeling events described during evolutionarily conserved stress adaptation [31,32,38,39], we investigated whether their formation reflected activation of the canonical unfolded protein response. Notably, robust activation of the UPR by tunicamycin, thapsigargin, and dithiothreitol failed to induce detectable p53–CerS6 interaction despite strong activation of canonical UPR markers. Conversely, metabolic stress resulted in formation of p53–CerS6 complexes, with little or no activation of the canonical UPR. Because the canonical UPR activation occurred significantly earlier than BiFC (before 4 h) yet failed to promote p53–CerS6 interaction, these data argue that assembly of the CerS6–p53 complex is not a downstream consequence of UPR signaling under the conditions examined but instead represents a mechanistically distinct metabolic stress response. This conclusion is particularly interesting in light of previous studies demonstrating that CerS6 can influence ER stress signaling through the ATF6/CHOP pathway [29]. Rather than contradicting those findings, our results suggest that CerS6 participates in multiple context-dependent stress-response pathways, with p53 recruitment representing a specialized signaling output that is independent of canonical UPR activation.

The selective nature of p53–CerS6 complex formation further emphasizes that this pathway is governed by stimulus-specific signaling rather than by p53 activation alone. Although several agents that activate p53, including MTX, PyrC_16_, gemcitabine, doxorubicin, and Nutlin-3, promoted robust p53–CerS6 interaction, other well-established p53-activating stimuli, including actinomycin D, daunorubicin, etoposide, and the proteasome inhibitor MG-132, failed to do so under the conditions examined. The inability of MG-132 to induce p53–CerS6 interaction is particularly informative because this agent stabilizes p53 through inhibition of proteasomal degradation [40,41] yet does not promote recruitment of p53 to CerS6. Thus, accumulation of p53 alone is insufficient for complex formation, indicating that additional upstream signaling events are required. Likewise, the differential responses observed among agents targeting the same pathways, such as MTX and lometrexol, or doxorubicin and daunorubicin, suggest that activation of the CerS6–p53 pathway depends not simply on broad classifications such as metabolic stress or DNA damage, but rather on specific cellular contexts that remain to be defined. Collectively, our findings indicate that recruitment of p53 to CerS6 is governed by a highly selective signaling mechanism that cannot be explained solely by broad stimulus categories such as metabolic stress, DNA damage, MDM2 inhibition, or p53 stabilization. Identification of the upstream signals that specify CerS6–p53 complex formation represents an important direction for future investigation.

Our findings expand the current paradigm of p53 regulation by identifying direct lipid recognition as a previously unappreciated mechanism through which metabolic state can influence tumor suppressor activity. Classically, p53 activity is viewed as being controlled primarily through post-translational modifications and protein–protein interactions [33,42]. Our findings support a model in which lipid signaling also contributes directly to p53 activation. This mechanism provides an efficient means of coupling changes in cellular metabolism to the p53 stress response, allowing metabolic perturbations to be translated into transcriptional programs governing cell-cycle arrest and apoptosis [33]. The observation that several common cancer-associated *TP53* mutants retain C_16_-ceramide recognition further raises the possibility that this regulatory interface remains accessible in a subset of human tumors despite the loss of canonical p53 function. Although the present study does not establish whether enhancing ceramide binding can restore tumor suppressor activity in mutant p53 proteins, the existence of a conserved lipid-recognition surface suggests new opportunities for therapeutic strategies aimed at modulating p53 through metabolic signaling rather than direct restoration of DNA binding [37].

Despite defining the structural and functional importance of the C_16_-ceramide-binding surface, several mechanistic questions remain unresolved. Cryo-EM and X-ray crystallographic studies of the p53 DBD in complex with C_16_-ceramide will be important for defining the molecular basis of lipid recognition. Likewise, the mechanism by which CerS6 transfers newly synthesized C_16_-ceramide to p53 at the cytosolic face of the endoplasmic reticulum remains to be elucidated. An important unanswered question is how diverse cellular stresses are integrated to promote selective recruitment of p53 to CerS6. Defining these upstream signals and determining how they influence localized ceramide production, p53 recruitment, and complex assembly will be important for understanding the selectivity of p53 activation by metabolic stress.

## 5. Conclusions

In summary, our study defines structural determinants of the previously identified C_16_-ceramide-binding surface of p53 and demonstrates a strong association between ceramide-binding competence, stress-induced p53–CerS6 complex formation, and downstream p53 signaling. Identification of a ceramide-binding regulatory surface within the p53 DBD, together with preservation of this interaction in multiple cancer-associated p53 mutants, expands our understanding of how metabolic stress is communicated to the p53 pathway.

Together, these findings support a model in which CerS6 serves not only as the biosynthetic source of C_16_-ceramide [43] but also as a spatial organizer that facilitates localized lipid transfer to p53, thereby establishing direct lipid recognition as a previously unrecognized mechanism linking specific metabolic stress to tumor suppressor activation.

## Figures and Tables

**Table 1:** Immunoblotting conditions.

Target	Protein Loaded (µg)	Gel (%)	Transfer (min)	Blocking Buffer	Primary Dilution	Secondary Dilution
p53 (IH)	30	12	80	3% Milk	1:3000	1:5000
CerS6	15	12	80	3% Milk	1:3000	1:5000
CHOP	30	12	80	3% BSA	1:500	1:2000
p-eIF2α	30	12	80	3% BSA	1:500	1:2000
BiP	20	10	80	3% BSA	1:10,000	1:15,000
ATF6	30	10	80	3% Milk	1:500	1:5000
p-IRE1α	30	10	110	3% BSA	1:500	1:2000
p21	30	15	70	3% Milk	1:500	1:2000
PUMA	30	15	70	3% BSA	1:500	1:2000
β-actin (HRP)	—	Same gel	—	—	1:20,000	—

## Data Availability

The original contributions presented in this study are included in the article/Supplementary Materials. Further inquiries can be directed to the corresponding author.

## Supplementary Materials

The following supporting information can be downloaded at https://www.mdpi.com/article/10.3390/biom16091343/s1, Figure S1. Validation of p53 mutant expression and ceramide-binding assays. Figure S2. Complete BiFC analysis of p53 mutants. Figure S3. Expanded functional analysis of p53 ceramide-binding mutants. Figure S4. Expanded analysis of cancer-associated p53 mutants. Figure S5. Supporting information for Figure 5 immunoblot analyses. Figure S6. ER co-localization of p53–CerS6 BiFC induced by distinct cellular stressors. Table S1. Antibodies Used in this Study.

## Abbreviations

The following abbreviations are used in this manuscript:

ATF6	Activating Transcription Factor 6
BiFC	Bimolecular Fluorescence Complementation
BiP	Binding Immunoglobulin Protein
CalR	Calreticulin
CerS6	Ceramide Synthase 6
CHOP	C/EBP Homologous Protein
DBD	DNA-Binding Domain
DTT	Dithiothreitol
ER	Endoplasmic Reticulum
MDM2	Mouse Double Minute 2 Homolog
MTX	Methotrexate
PyrC_16_	Pyridinium C_16_-ceramide
PyrC_6_	Pyridinium C_6_-ceramide
TG	Thapsigargin
TM	Tunicamycin
UPR	Unfolded Protein Response
V1	N-Terminal Fragment of Venus Protein
V2	C-Terminal Fragment of Venus Protein
WT	Wild-type
