# Supplementary material for: Determinants of C16-Ceramide Binding to p53
Source: Biomolecules. 2026 Sep 15;16(9):1343. doi: 10.3390/biom16091343 (PMC13604752; doi:10.3390/biom16091343)
Supplement: Supplementary file 1 [file biomolecules-16-01343-s001.zip › biomolecules-4483528-supplementary.pdf]

Supplemental Figure S1

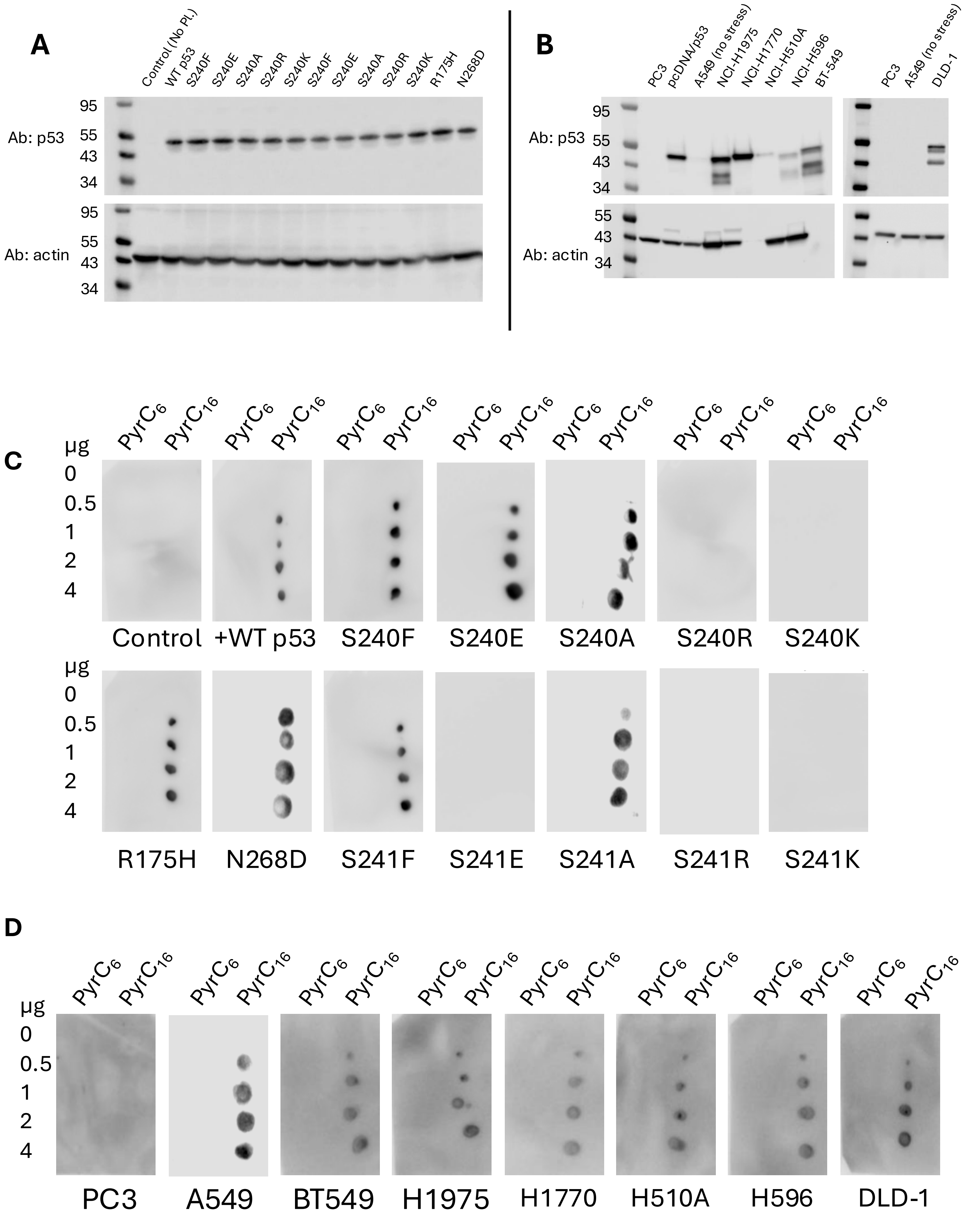

Supplemental Figure S1 Validation of p53 mutant expression and ceramide-binding assays.

(A) Immunoblot confirming expression of WT and mutant p53 proteins following transient transfection in A549/p53-shRNA cells. Actin served as a loading control. **(B)** Endogenous p53 expression in the cell lines used in this study. Actin served as a loading control**. (C)** Complete dot blot analyses corresponding to **Fig. 1B** of WT p53 and all p53 mutants tested for binding to $PyrC_{16}$ and the negative control lipid $PyrC_6$. All analyses were performed in triplicate ,and representative experiments are shown. **(D)** Complete dot blot analyses corresponding to **Fig. 4C** of endogenous p53 from the indicated cell lines demonstrating selective binding to $PyrC_{16}$ and the negative control lipid $PyrC_6$. All analyses were performed in triplicate, and representative experiments are shown.

Supplemental Figure S2

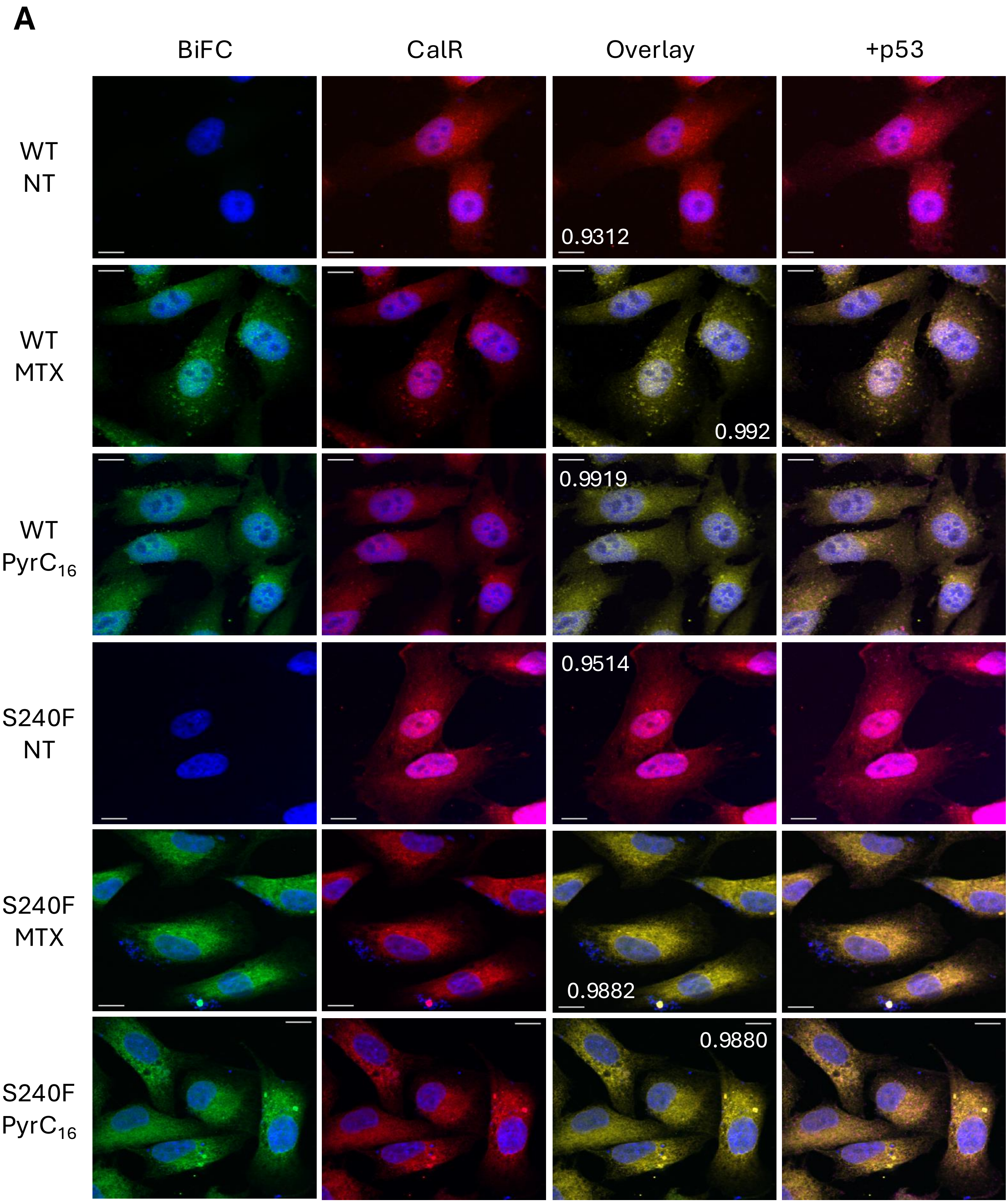

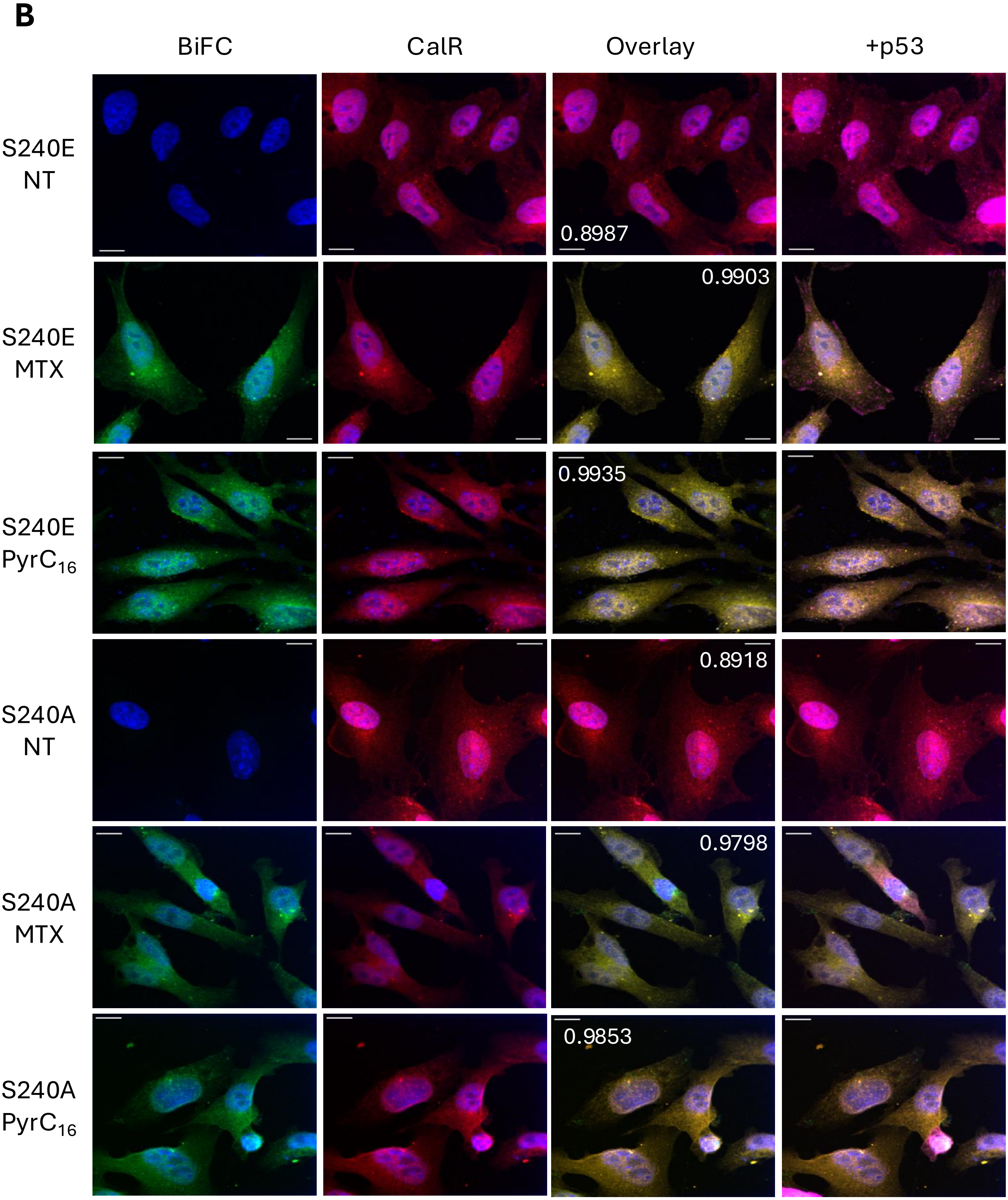
B
BiFC
CalR
Overlay
+p53
S240E NT
0.8987
S240E MTX
0.9903
S240E PyrC16
0.9935
S240A NT
0.8918
S240A MTX
0.9798
S240A PyrC16
0.9853

C

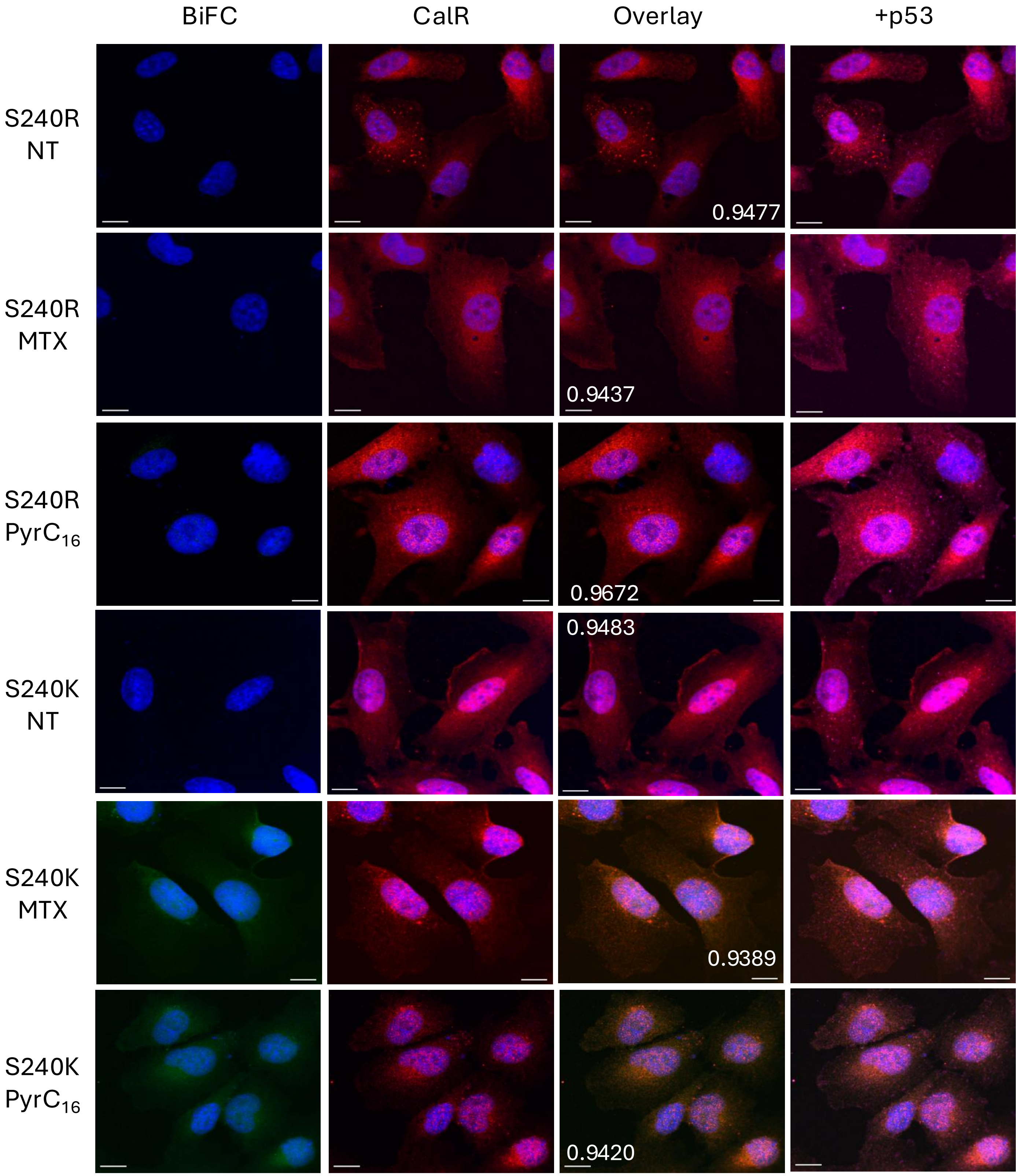

BiFC
CalR
Overlay
+p53
S240R NT
0.9477
S240R MTX
0.9437
S240R PyrC16
0.9672
S240K NT
0.9483
S240K MTX
0.9389
S240K PyrC16
0.9420

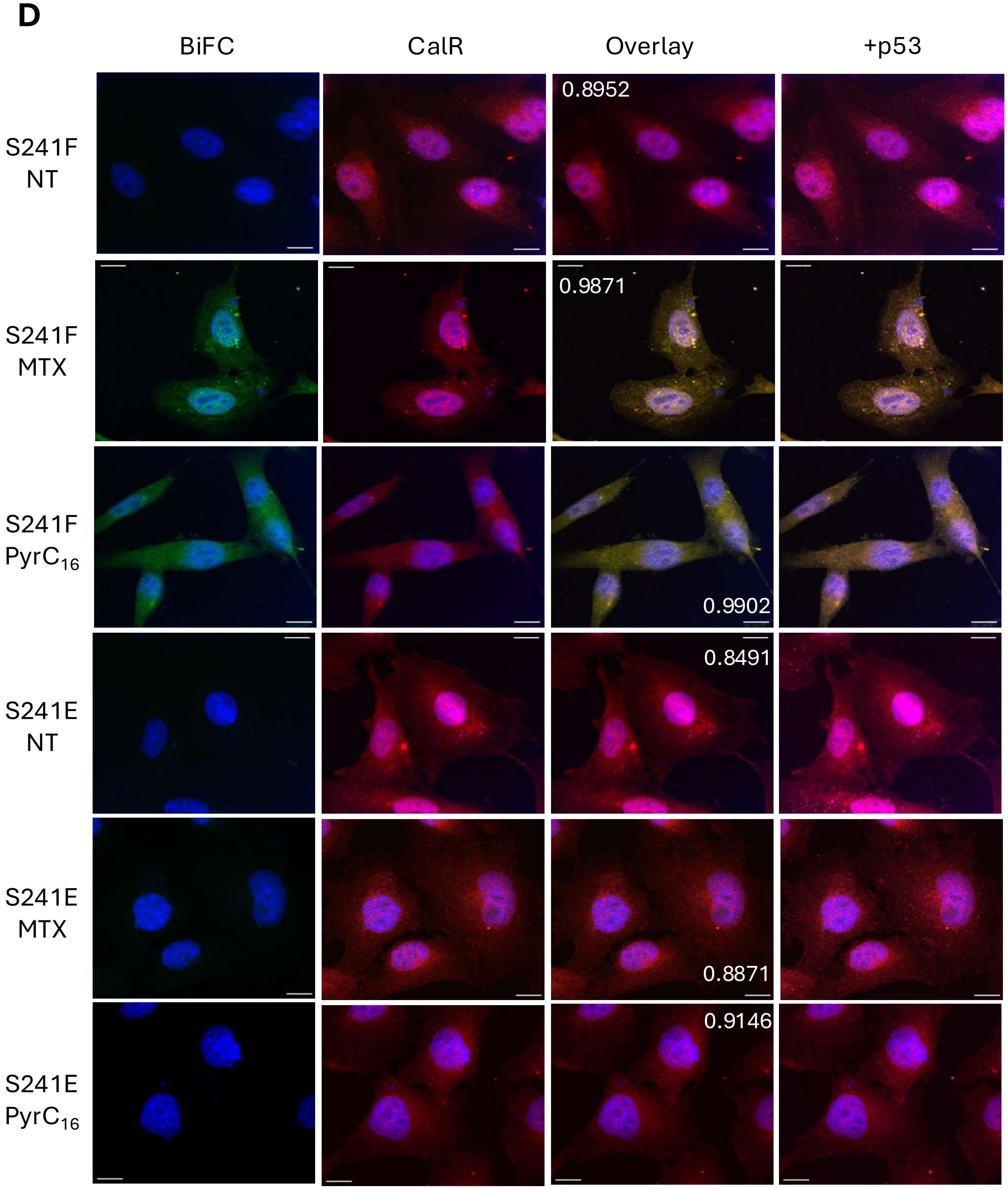
D
BiFC
CalR
Overlay
+p53
S241F NT
0.8952
S241F MTX
0.9871
S241F PyrC$_{16}$
0.9902
S241E NT
0.8491
S241E MTX
0.8871
S241E PyrC$_{16}$
0.9146

E

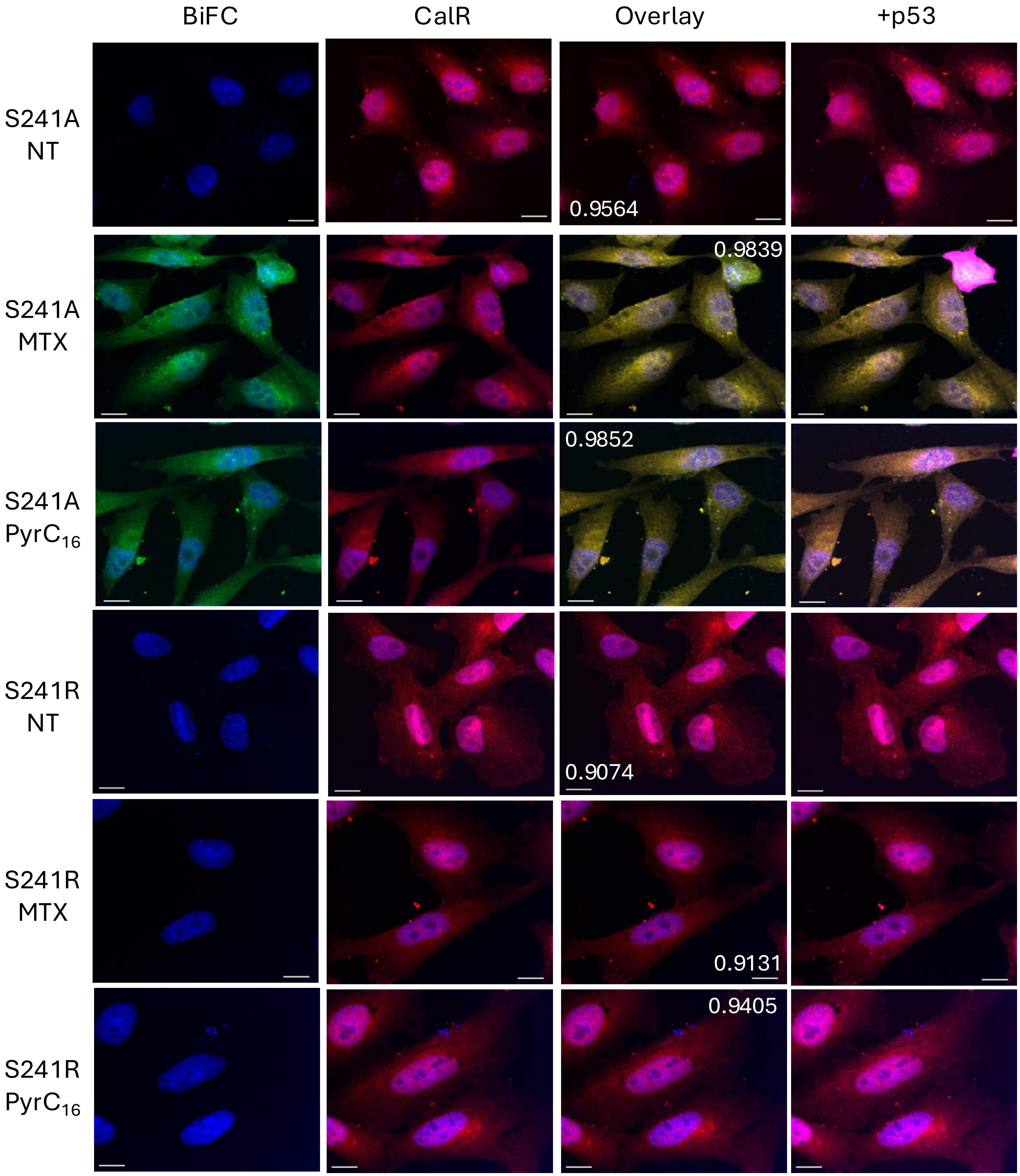

BiFC
CalR
Overlay
+p53
S241A NT
S241A MTX
S241A PyrC16
S241R NT
S241R MTX
S241R PyrC16
0.9564
0.9839
0.9852
0.9074
0.9131
0.9405

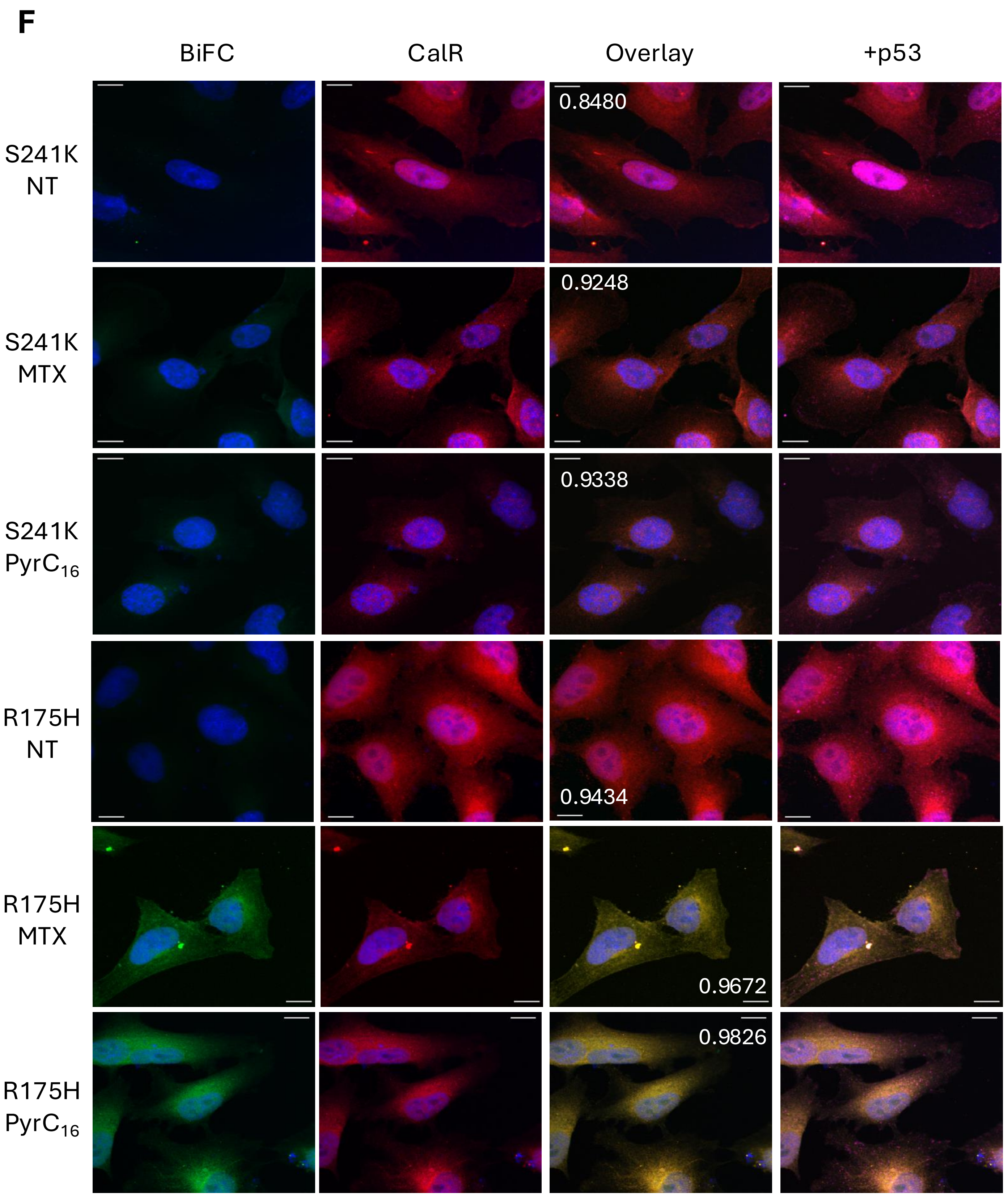
F
BiFC
CalR
Overlay
+p53
S241K NT
0.8480
S241K MTX
0.9248
S241K PyrC16
0.9338
R175H NT
0.9434
R175H MTX
0.9672
R175H PyrC16
0.9826

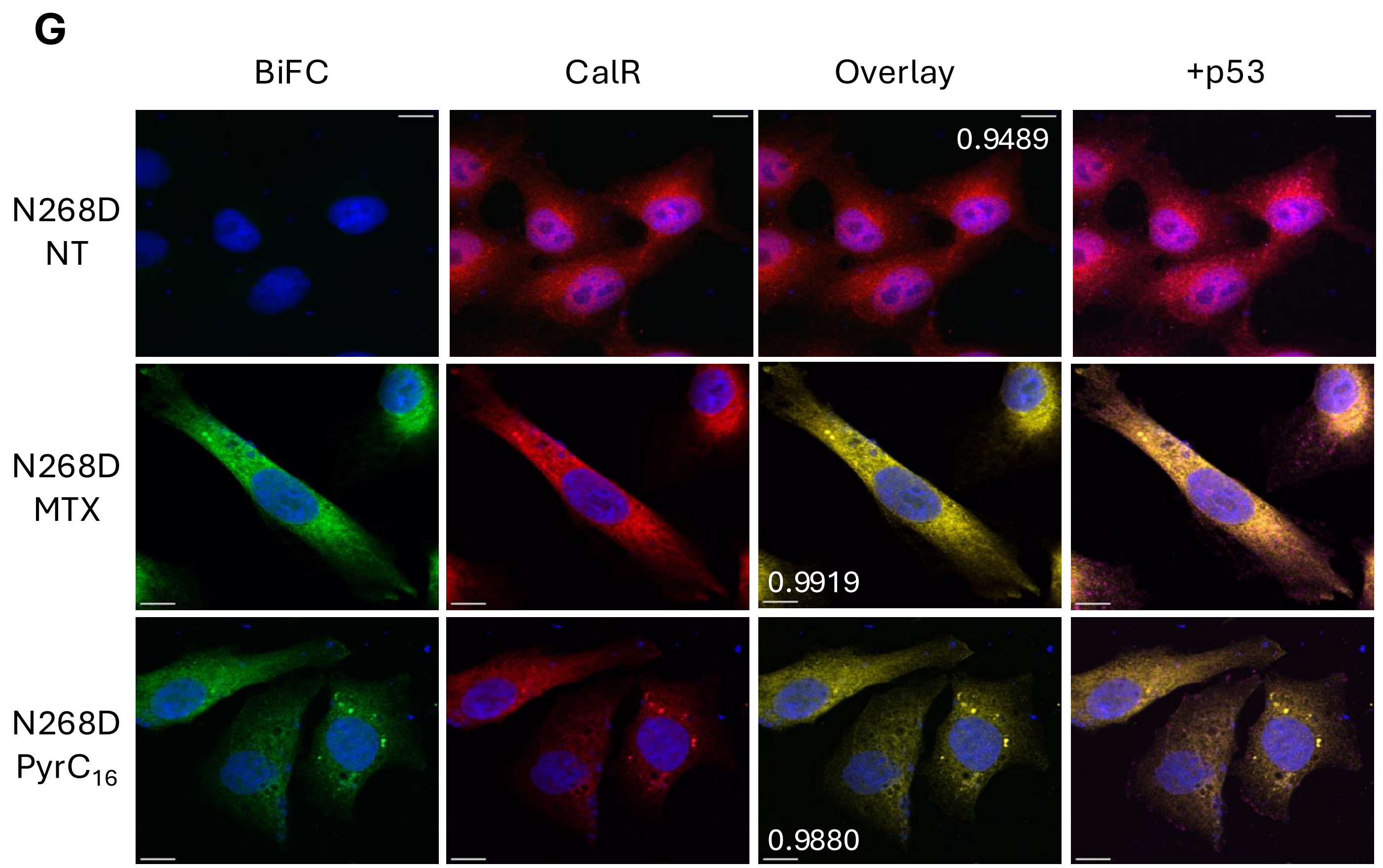
G
BiFC
CalR
Overlay
+p53
N268D NT
N268D MTX
N268D PyrC16
0.9489
0.9919
0.9880

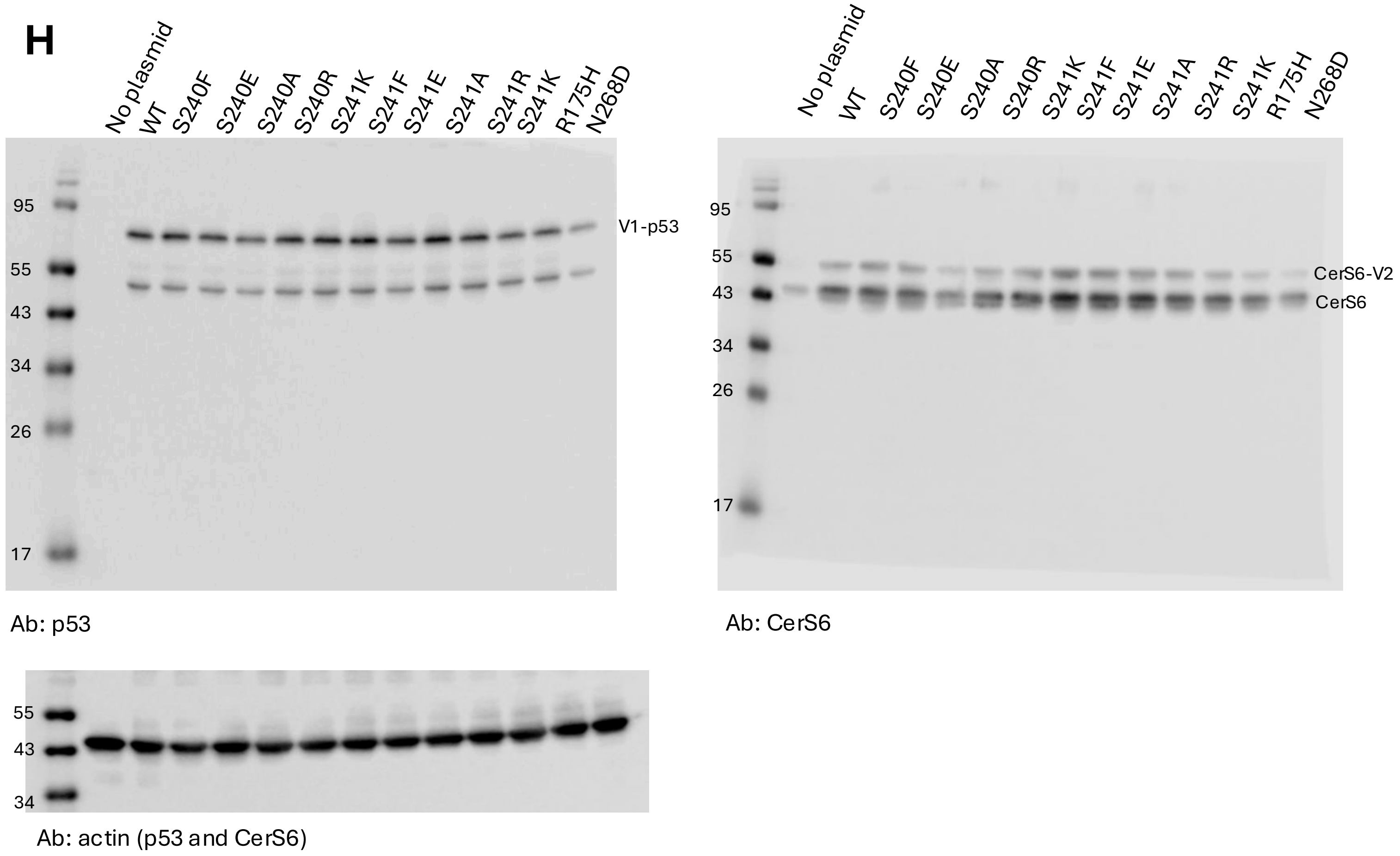
H
No plasmid
WT
S240F
S240E
S240A
S240R
S241K
S241F
S241E
S241A
S241R
S241K
R175H
N268D
95
55
43
34
26
17
V1-p53
CerS6-V2
CerS6
Ab: p53
Ab: CerS6
Ab: actin (p53 and CerS6)

Supplemental Figure S2 Complete BiFC analysis of p53 mutants.

(A) – (G) Representative BiFC images for all p53 mutants under NT, MTX (10 nM, 24 h), and $PyrC_{16}$ (5 µM, 24 h) treatment. Venus fluorescence (green) indicates p53–CerS6 interaction, CalR (red) marks the ER, DAPI (blue) labels nuclei, and yellow indicates co-localization of BiFC and ER signals in merged images. All analyses were performed in triplicate, and representative experiments are shown. Pearson's correlation coefficients are shown. (+p53) Corresponding p53 immunofluorescence. Scale bars, 20 µm. 140× magnification. **(H)** Immunoblot confirmes expression of V1-p53 and CerS6-V2 constructs used for BiFC analyses. Actin served as a loading control. Analysis were performed in triplicate, and representative experiments are shown.

Supplemental Figure S3

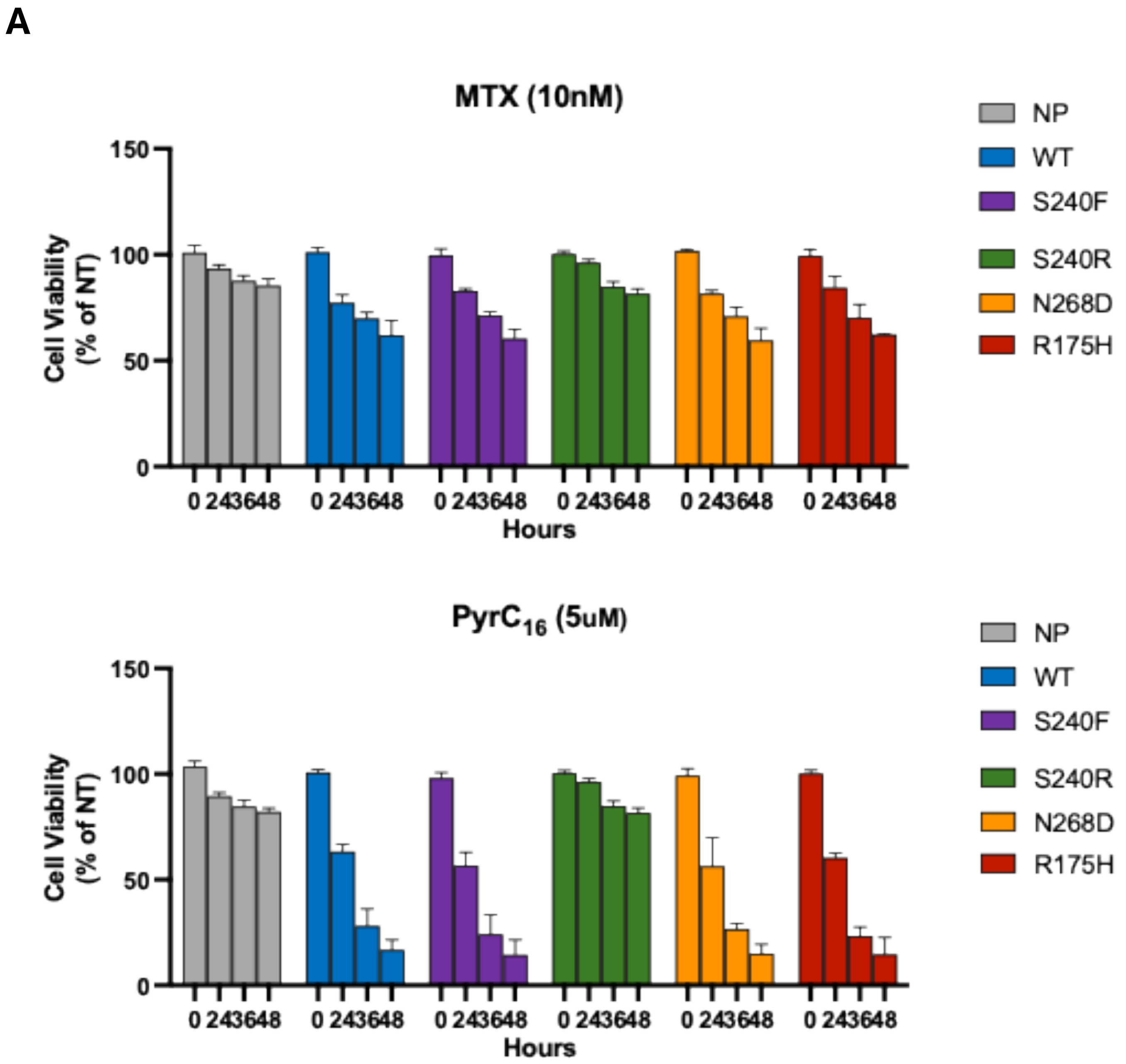

Supplemental Figure S3

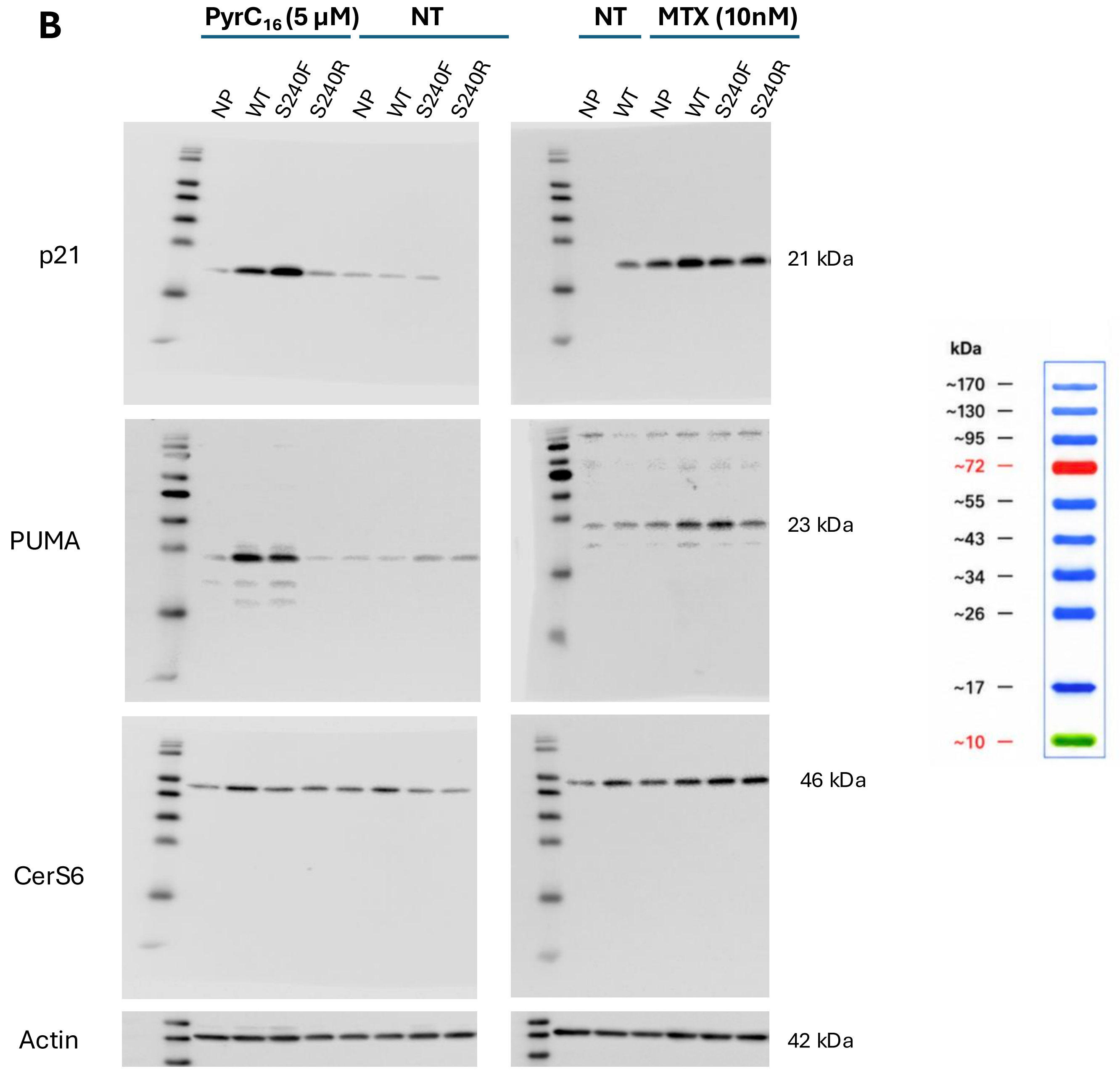

Supplemental Figure S3 Expanded functional analysis of p53 ceramide-binding mutants.

(A) Time-course MTT analysis of p53 mutants following treatment with MTX (10 nM) or $PyrC_{16}$ (5 μM). NP, no plasmid. All analyses were performed in triplicate, with the representative experiments shown. **(B)** Uncropped immunoblots corresponding to **Fig. 3** showing p21, PUMA, CerS6, and actin expression following NT, MTX, or $PyrC_{16}$ treatment.

Supplemental Figure S4

A

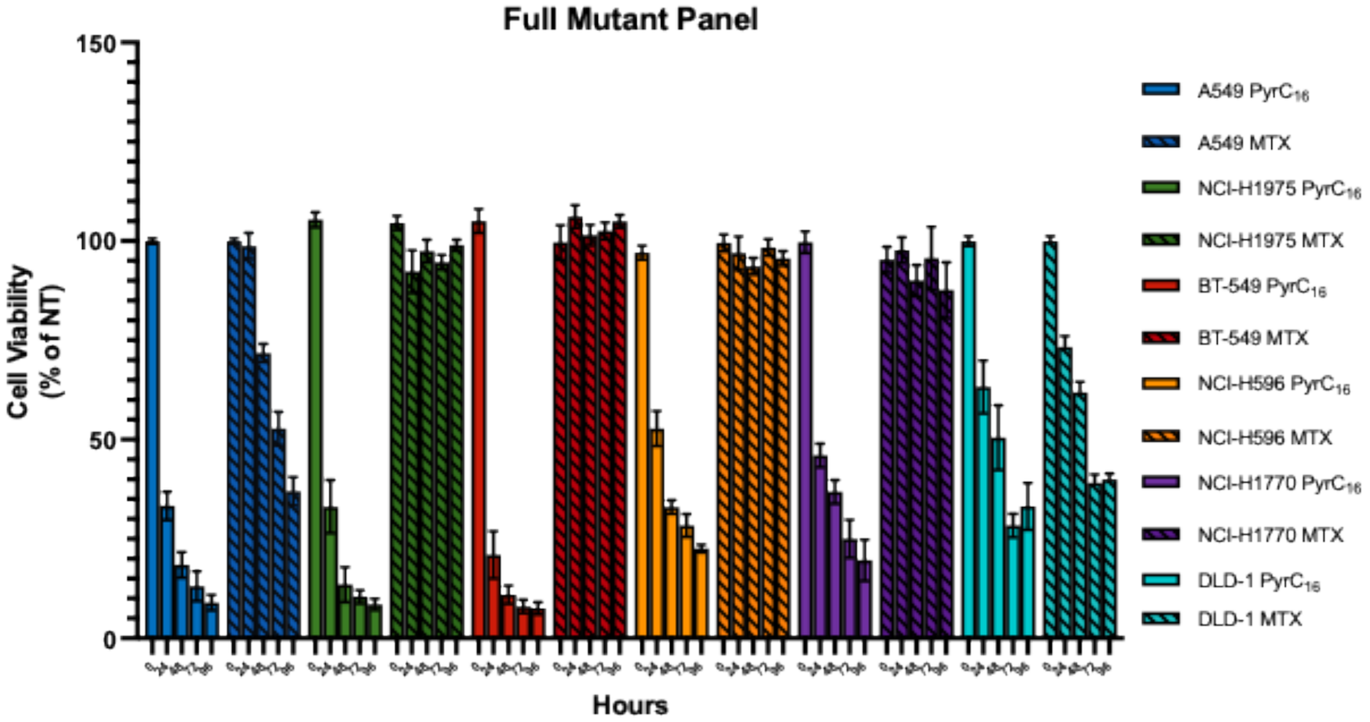

B

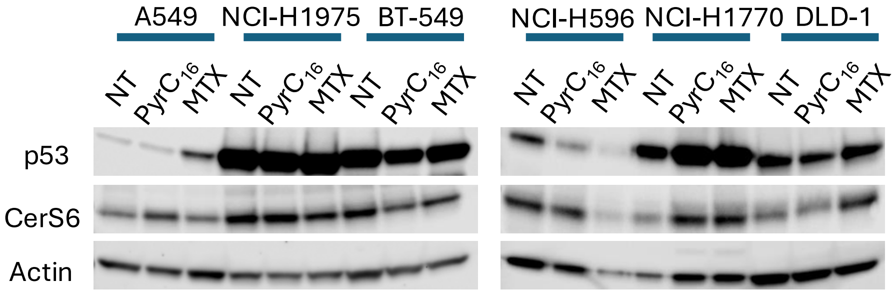

Supplemental Figure S4 Expanded analysis of cancer-associated p53 mutants.

(A) Cell viability analysis of the complete panel of cancer-associated p53 mutant cell lines following MTX or $PyrC_{16}$ treatment. **(B)** Representative immunoblots of endogenous p53 and CerS6 expression following NT, MTX, or $PyrC_{16}$ treatment in each cell line. Actin served as a loading control.

Supplemental Figure S5

A Master Lane Layout (Applies to all blots below)

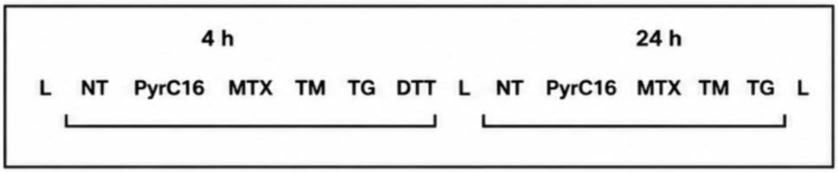

B Molecular Weight Reference

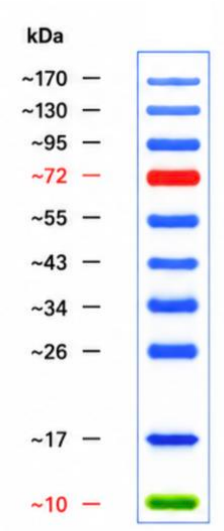

Protein	Expected MW (kDa)
p53	53
p21	21
PUMA	23
CerS6	44
CHOP	30
BiP	78
p-eIF2α	38
p-IRE1α	110
ATF6-FL	90
ATF6-N	50
Actin	42

C **Uncropped Western Blots: A549 WT**

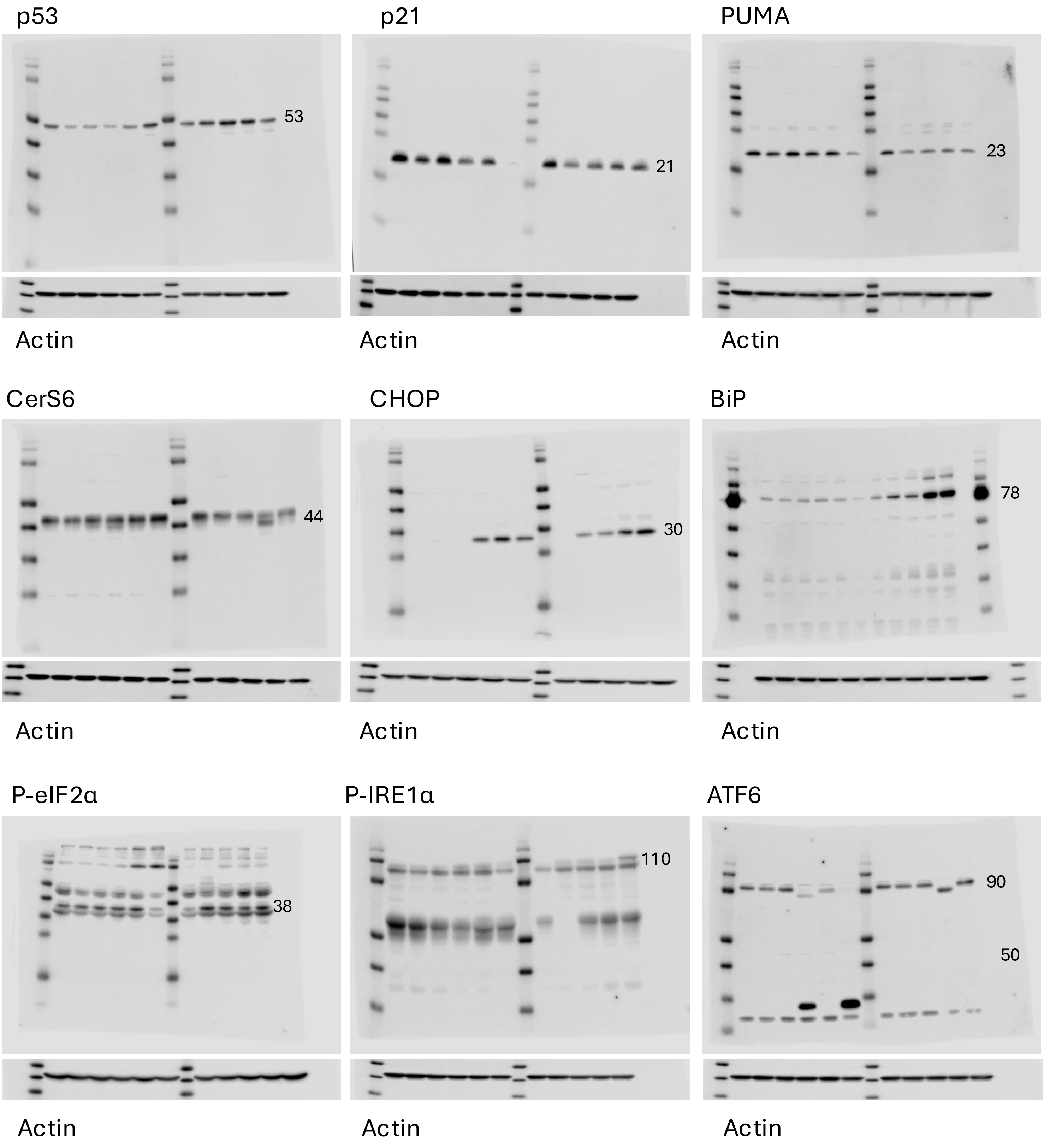

D **Uncropped Western Blots: A549 $p53^{-/-}$**

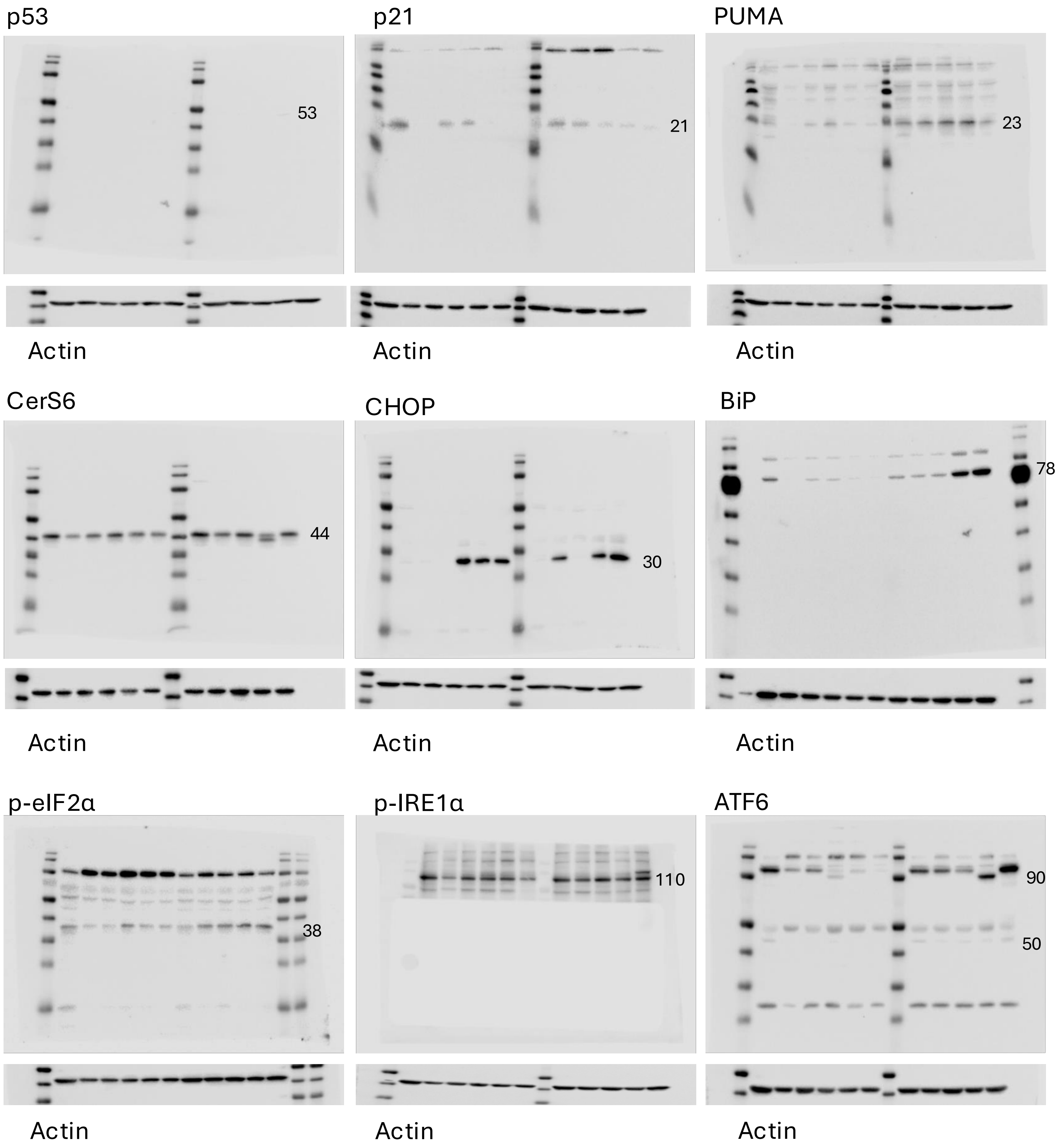

Supplemental Figure S5 Supporting information for Fig. 5 immunoblot analyses.

(A) Master lane layout used for all immunoblots shown in **Fig. 5**. **(B)** Molecular weight reference and expected migration of proteins analyzed. **(C)** Uncropped immunoblots from A549 WT cells. **(D)** Uncropped immunoblots from A549/p53-shRNA cells. Actin loading controls corresponding to each blot are shown.

Supplemental Figure S6

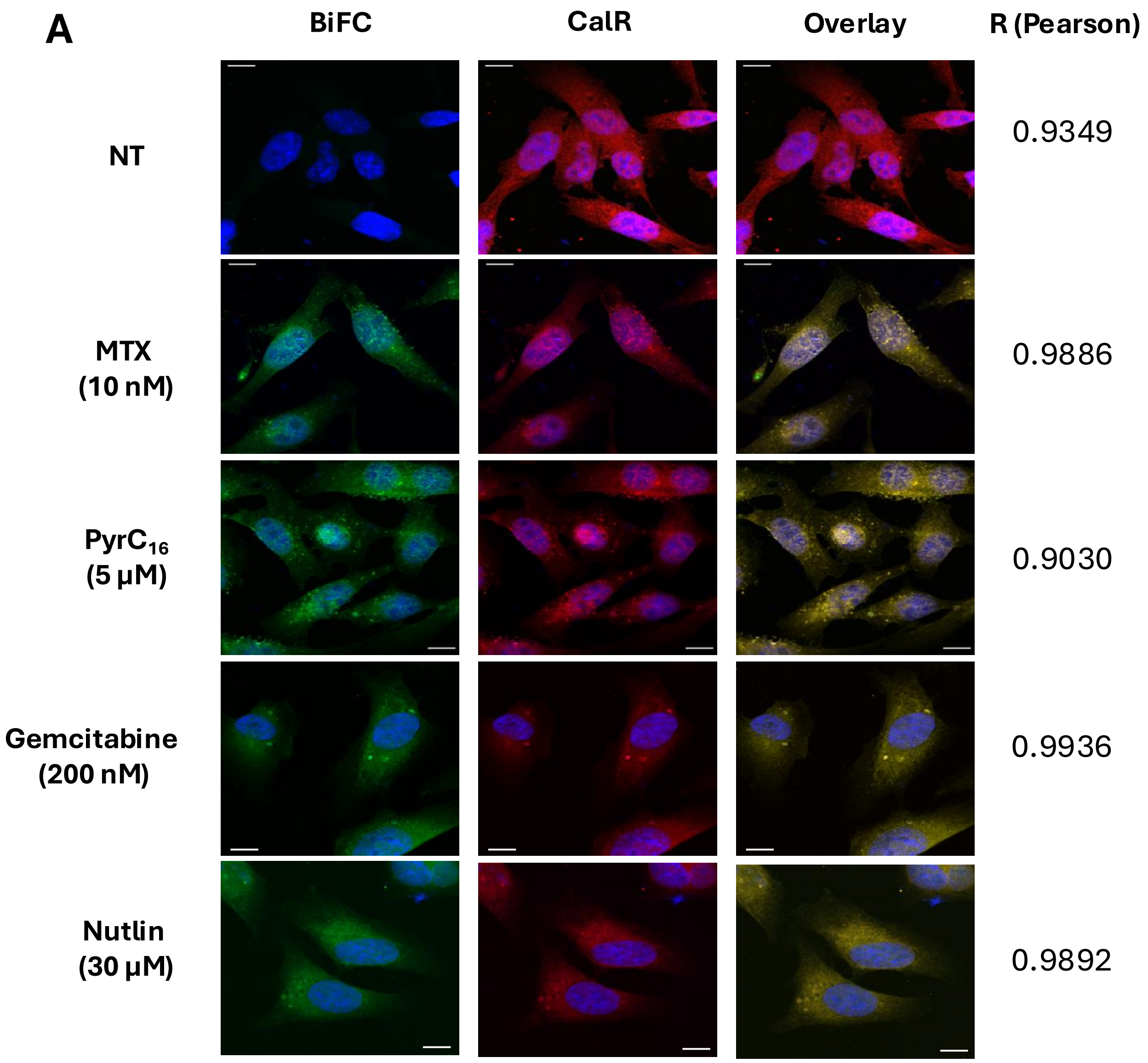

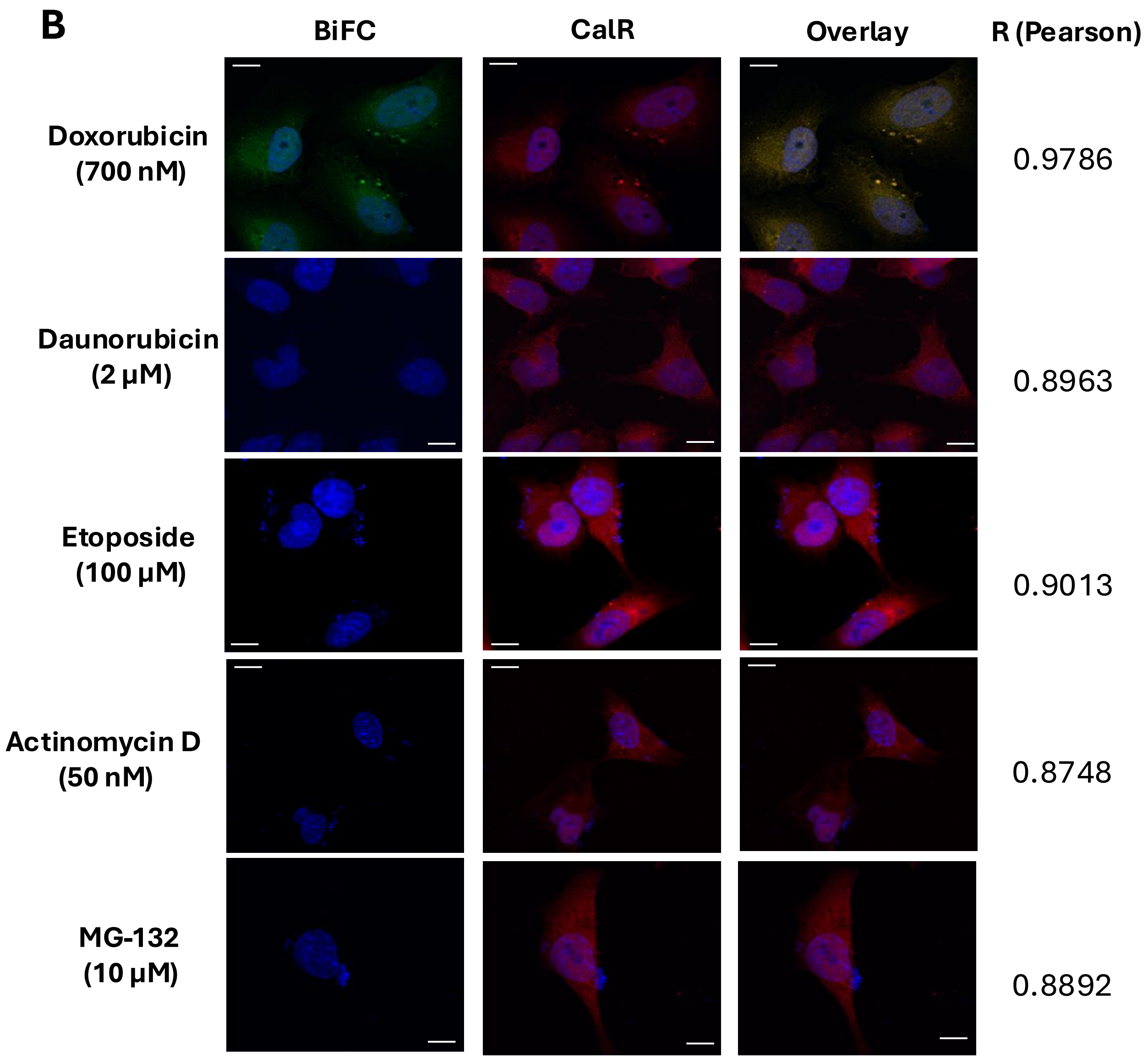
B
BiFC
CalR
Overlay
R (Pearson)
Doxorubicin (700 nM)
0.9786
Daunorubicin (2 µM)
0.8963
Etoposide (100 µM)
0.9013
Actinomycin D (50 nM)
0.8748
MG-132 (10 µM)
0.8892

Supplemental Figure S6 ER co-localization of p53–CerS6 BiFC induced by distinct cellular stressors. (A) & (B) Representative BiFC images corresponding to **Fig. 6** showing CalR staining (*red*), BiFC fluorescence (*green*), and merged images. Yellow indicates co-localization, and Pearson's correlation coefficients for the indicated treatments are shown on in overlay panels. DAPI (*blue*) labels nuclei. Scale bars, 20 μm. 140× magnification.

Supplemental Table S1

	Host	Supplier	Catalog No.	Application	Dilution
**p53 (IH)**	Rabbit	In-house	—	WB/MB	1:3000
**p53**	Mouse	Santa Cruz Biotechnology	sc-126	IF	1:300
**Calreticulin**	Rabbit	Invitrogen	PA3-900	IF	1:300
**CerS6**	Rabbit	In-house	—	WB	1:3,000
**CHOP**	Rabbit	Cell Signaling	#5554	WB	1:500
**p-eIF2α**	Rabbit	Cell Signaling	9721S	WB	1:500
**BiP**	Rabbit	Cell Signaling	#3183	WB	1:10,000
**ATF6**	Rabbit	Abcam	AB37149	WB	1:500
**p-IRE1α**	Rabbit	Invitrogen	PA116927	WB	1:500
**p21**	Rabbit	Cell Signaling	#2947	WB	1:500
**PUMA**	Rabbit	Cell Signaling	#12450	WB	1:500
**β-actin (HRP-conjugated)**	Mouse	Abcam	ab49900	WB	1:20,000
**Donkey anti-rabbit HRP**	Donkey	GE Healthcare	NA934	WB	1:2000<br>1:5000
**Donkey anti-rabbit Alexa Fluor 555**	Donkey	Invitrogen	A31572	IF	1:300
**Goat anti-mouse Alexa Fluor 568**	Goat	Invitrogen	A11031	IF	1:300
**Chicken anti-mouse Alexa Fluor 647**	Chicken	Invitrogen	A21463	IF	1:300

Supplemental Table S1 Antibodies Used in this Study.
